# Integrative chromosome-level genomics and metabolomics uncover regulatory networks linking monoterpenoid biosynthesis and glandular trichome formation in *Mosla chinensis*

**DOI:** 10.1093/hr/uhaf263

**Published:** 2025-10-01

**Authors:** Muyao Yu, Chenyi Li, Xiaoqing Wang, Dan Jiang, Xueqing Fu, Chao Chen, Guangxi Ren, Xuewei Li, Yaojie Zhang, Qi Liu, Shuyi Qian, Yang Han, Xiaoqun He, Zhenfang Bai, Badalahu Tai, Luqi Huang, Jinbao Yu, Han Zheng, Chunsheng Liu

**Affiliations:** School of Chinese Materia Medica, Beijing University of Chinese Medicine, Beijing 102488, China; Department of Biochemistry, Institute of Plant Biology, School of Life Sciences, Fudan University, Shanghai 200438, China; Key Laboratory of Germplasm Selection and Breeding of Chinese Medicinal Materials, Jiangxi Provincial Institute of Traditional Chinese Medicine, Nanchang 330046, China; School of Chinese Materia Medica, Beijing University of Chinese Medicine, Beijing 102488, China; School of Agriculture and Biology, Shanghai Jiao Tong University, Shanghai 200240, China; Key Laboratory of Germplasm Selection and Breeding of Chinese Medicinal Materials, Jiangxi Provincial Institute of Traditional Chinese Medicine, Nanchang 330046, China; School of Chinese Materia Medica, Beijing University of Chinese Medicine, Beijing 102488, China; Key Laboratory of Germplasm Selection and Breeding of Chinese Medicinal Materials, Jiangxi Provincial Institute of Traditional Chinese Medicine, Nanchang 330046, China; School of Agriculture and Biology, Shanghai Jiao Tong University, Shanghai 200240, China; State Key Laboratory for Quality Ensurance and Sustainable Use of Dao-di Herbs, National Resource Center for Chinese Materia Medica, China Academy of Chinese Medical Sciences, Beijing 100700, China; School of Agriculture and Biology, Shanghai Jiao Tong University, Shanghai 200240, China; State Key Laboratory for Quality Ensurance and Sustainable Use of Dao-di Herbs, National Resource Center for Chinese Materia Medica, China Academy of Chinese Medical Sciences, Beijing 100700, China; Key Laboratory of Germplasm Selection and Breeding of Chinese Medicinal Materials, Jiangxi Provincial Institute of Traditional Chinese Medicine, Nanchang 330046, China; School of Chinese Materia Medica, Beijing University of Chinese Medicine, Beijing 102488, China; State Key Laboratory for Quality Ensurance and Sustainable Use of Dao-di Herbs, National Resource Center for Chinese Materia Medica, China Academy of Chinese Medical Sciences, Beijing 100700, China; State Key Laboratory for Quality Ensurance and Sustainable Use of Dao-di Herbs, National Resource Center for Chinese Materia Medica, China Academy of Chinese Medical Sciences, Beijing 100700, China; Key Laboratory of Germplasm Selection and Breeding of Chinese Medicinal Materials, Jiangxi Provincial Institute of Traditional Chinese Medicine, Nanchang 330046, China; School of Agriculture and Biology, Shanghai Jiao Tong University, Shanghai 200240, China; State Key Laboratory for Quality Ensurance and Sustainable Use of Dao-di Herbs, National Resource Center for Chinese Materia Medica, China Academy of Chinese Medical Sciences, Beijing 100700, China; School of Chinese Materia Medica, Beijing University of Chinese Medicine, Beijing 102488, China

## Abstract

Xiangru, with *Mosla chinensis* (Mc, 2*n* = 18) and its considered cultivar *M. chinensis* ‘Jiangxiangru’ (McJ, 2*n* = 18) as original plants, is an annual herb of the Lamiaceae family, and is widely used as medicinal and edible plant due to its spleen strengthening function. However, absence of genomic resource impedes in-depth research towards Xiangru. In this study, the morphological characteristics and volatile organic compounds (VOC) contents of Mc and McJ were analyzed, showing higher trichome density and monoterpenoid accumulation obtained in Mc, whereas McJ possessed higher biomass. We assembled high-quality Mc, McJ, and their adulterant *Mosla soochowensis* (2*n* = 18) genomes of 426.1, 408.8, and 412.8 Mb, respectively, containing the repeat sequences of 57.17%, 56.33%, and 55.83%. Comparative genomics analysis indicated *Mosla* radiated ~13.3 Mya, supporting McJ initially as a natural naturally formed resource. Five monoterpene synthase genes were identified through comparative transcriptome and were responsible for catalyzing production of diversified monoterpene skeleton, in which TPS1 mediated formation of γ-terpinene, accompanied by CYP71D179 and SDR2, leading to the final production of carvacrol and thymol. We further explored correlation between monoterpenoids biosynthesis and trichome development, indicating MIXTA and WIN1 jointly regulate both trichome formation and VOC accumulation by directly binding promoters of *TPS1* and *CYP71D179*, respectively. Our study fills vacancy of genus *Mosla* genomes, improving the biosynthetic and regulatory mechanism of volatile compounds in aromatic Traditional Chinese Medicine, also offering novel targets for quality-directed breeding in Xiangru.

## Introduction

*Mosla chinensis* Maxim. (Mc) and its considered cultivated variant *M. chinensis* ‘Jiangxiangru’ (McJ), the botanical origins of the traditional Chinese medicine Xiangru, have been used for over a millennium for their antipyretic and analgesic properties. Historical records trace the medicinal use of wild Mc to the Song dynasty (ca. 973 AD), while its subsequent domestication likely led to McJ as a high-yielding resource—now the primary commercial source of Xiangru [1]. However, the inadvertent cultivation of *Mosla soochowensis* (Ms), a phylogenetically related but taxonomically distinct species (Fig. S1), has complicated the authentication of Xiangru materials. Critically, despite its medicinal importance, the *Mosla* genus (Lamiaceae: Nepetoideae, Elsholtzieae) remains genomically unexplored [2], hindering both research into Xiangru’s pharmacodynamic basis and broader studies of Lamiaceae evolution.

The therapeutic effects of Xiangru are attributed to volatile organic compounds (VOCs), notably carvacrol, thymol, cineole, linalool, etc., which exhibit anti-inflammatory, antiviral, and immunomodulatory activities. In related Lamiaceae species, thymol and carvacrol biosynthesis begins with the oxidation of γ-terpinene, in which terpene synthase (TPS) catalyze the cyclization, such as OvTPS2 in *Origanum vulgare* [3], TcTPS2 in *Thymus caespititius* [4], TvTPS2 in *Thymus vulgaris* [5], and TqTPS1 in *Thymus quinquecostatus* [6]. Subsequently, the aromatic backbone is catalyzed by cytochrome P450 monooxygenases (CYPs) in combination with a short-chain dehydrogenase (SDR) via an unstable intermediate in *T. vulgaris* [5]. Yet, the evolutionary origins and regulatory mechanisms of these pathways in *Mosla* remain unresolved, impeding efforts to optimize its medicinal quality.

Plant trichomes, specialized epidermal structures on stems, leaves, and flowers, serve as primary sites for the synthesis and storage of volatile monoterpenoids and sesquiterpenoids [7]. These trichomes are functionally classified into capitate glandular trichomes (CGTs), peltate glandular trichomes (PGTs), and nonglandular trichomes (NGTs). Notably, secretory trichomes (CGTs and PGTs) exhibit a well-documented positive correlation with terpene biosynthesis [8, 9], making trichome density manipulation a viable strategy for enhancing volatile compound production. Current understanding of trichome development largely stems from model systems such as *Solanum lycopersicum* and *Artemisia annua*, where phytohormone-mediated signaling cascades coordinate the actions of transcription factors (TFs), cell cycle regulators, and receptor proteins. Key regulators include the HD-ZIP family member Woolly, which promotes trichome initiation through interactions with HAIRs or CycB2 [10–12], and is itself modulated by MIXTA, JAZ2, and HD8 [13, 14]. Additional regulatory modules involve the MYB5-HD8-MYB108 axis [15, 16], SPL9 [17], and the JAZ8-SEP1-MYB16 cascade [18], while HD1 exerts direct or indirect control via TAR2 and GSW2 [19]. Further complexity arises from contributions by MYB1 [20], the TLR1-WOX1-TLR2 complex [21], the MYC1-TOR module [22], and C2H2 zinc finger proteins such as SAP1 [23] and ZFP6 [24]. Recent studies in *Mentha canadensis* and *Schizonepeta tenuifolia* have confirmed conserved roles for MIXTA [25] and HD1/HD8 [26] in trichome regulation. Despite these advances, the molecular mechanisms governing trichome development in *Mosla* species remain poorly characterized, representing a critical knowledge gap for targeted breeding of high-yield, high-quality medicinal varieties.

In this study, the reference genome sequences including Mc, its considered cultivated variant McJ, and its related species Ms have been reported through the use of a combination of high-fidelity (HiFi) sequencing and high-throughput chromatin conformation capture (Hi-C). Genome-scale sequencing data analysis, comparative genomics, phylogenetic, metabolomic, transcriptomic studies, *in vivo* verification, and transcription regulation verification were conducted to reveal evolutionary differences, bioactive monoterpenoids biosynthesis, and the unified regulation between trichome development and volatile metabolite accumulation in Xiangru, thereby providing genomic and metabolic resources that aid genetic function studies and molecular breeding of Mc and McJ.

## Results

### Different phenotypic and metabolomic characteristics obtained in Mc and McJ

**Figure 1:**
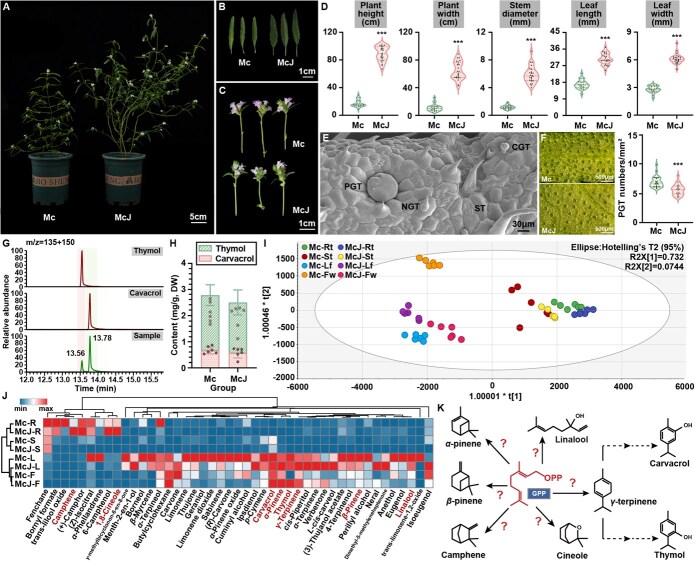
Phenotype and metabolome comparison between Mc and McJ. (A–C) Morphology including plant characteristics (A), leaf characteristics (B), and flower characteristics (C) of Mc and McJ. (D) Agronomic traits of Mc and McJ (*n* = 24). (E) Trichomes of Xiangru leaves. (F) PGT density of Mc and McJ (*n* = 24). (G) Chromatogram of thymol, carvacrol, and the sample. (H) Content of thymol and carvacrol in Mc and McJ (*n* = 6). (I) Multivariate statistical analysis of Mc and McJ metabolites. (J) Relative contents of monoterpenoids in roots, stems, leaves, and flowers of Mc and McJ. (K) Speculation on the formation of monoterpene skeletons in Xiangru. For box plots, data are presented as maximum, third quantile, median, first quartile, and minimum from top to bottom of the shape. For histogram, data are presented as mean ± SEM. Student’s *t*-test was performed for comparison, ^***^P < 0.001.

To study the characteristics of the two botanical origins of Xiangru, the wild Mc and cultivar McJ were collected in Jiangxi province and transplanted under identical conditions (Fig. 1A). Mc exhibited significantly longer, narrower leaves (Fig. 1B) and shorter inflorescence rachises (Fig. 1C) compared to McJ. Quantitative assessment of 24 biological replicates demonstrated that McJ showed 5.21-, 5.89-, 4.98-, 1.82-, and 2.18-fold increases in plant height, width, stem diameter, leaf length, and width, respectively (Fig. 1D, Table S1), confirming substantial morphological divergence between these two botanical origins.

Trichomes are specialized tissues from epidermal cells, also identifying features of Lamiaceae. Trichome features of Mc and McJ are consistent. As shown in Fig. 1E and Fig. S2A, NGTs and PGTs were mainly arranged on the abaxial surface of leaves. NGTs of Xiangru consist of one to seven cells, while the majority were one to three cells (Fig. S2A and B). PGT consisted of an ultrashort stalk and heads obtaining eight cells (Fig. 1E, Fig. S2C and D), while CGTs containing one- or two-celled stalks are occasionally seen (Fig. S2E). Notably, Mc displayed 1.24-fold higher trichome density than McJ (Fig. 1F, Table S2), correlating with its elevated production of key quality markers carvacrol (1.18-fold higher) and thymol (1.09-fold higher) as quantified by GC–MS, which was 1.18- and 1.09-fold higher than that in leaves of McJ, respectively (Fig. 1G and H, Table S3).

**Table 1:** Global statistics of Mc, McJ, and Ms assembly and annotation

**Parameter**	**Mc**	**McJ**	**Ms**
Chromosome numbers	9	9	9
Estimate of genome size, Mb	438.0	410.6	385.0
Assembled genome size, Mb	426.1	408.8	412.8
GC content, %	35.23	34.75	35.19
Number of contigs	65	51	135
N50 contig length, Mb	44.0	38.0	38.9
N50 scaffold length, Mb	44.8	47.3	-
Anchored scaffolds, %	96.7	97.2	-
Complete BUSCOs of genome, embryophyta_odb10, %	98.5	98.2	98.7
Repeat sequences, %	57.17	56.33	55.83
LAI	26.92	26.75	18.44
Number of protein-coding genes	32 297	33 245	34 133
Complete BUSCOs of proteins, embryophyta_odb10, %	99.0	98.2	98.8

To discover whether more different metabolites between Mc and McJ exist, volatile metabolomics analysis was performed on root, stem, leaf, and flower of Mc and McJ, identifying 197 differential metabolites, of which 45 were classified as monoterpenoids. Orthogonal partial least squares discriminant analysis (OPLS-DA) revealed distinct tissue-specific accumulation patterns, particularly between leaves–flowers and roots–stems (Fig. 1I). Hierarchical clustering demonstrated monoterpenoid enrichment preferably in leaves, followed by flowers and roots, and the least in stems. Additionally, compared with McJ, the content of monoterpenoids in Mc was relatively higher than that in McJ (Fig. 1J), consistent with the quantification results, indicating that the contents of volatile terpenoids positively correlated with the density of trichomes.

Additionally, it is worth noting that γ-terpinene, camphene, cineole, linalool, pinene, and other monoterpene parent nuclei, as well as many derivative compounds, appear in the metabolites, forming the structural basis of Xiangru’s characteristic volatile clusters. Thus, elucidating the mechanism of its evolution and biosynthesis will help to reveal the formation of pharmacodynamic material basis of Xiangru (Fig. 1K).

### Assembly and evolutionary analysis of the *Mosla* genome

**Figure 2:**
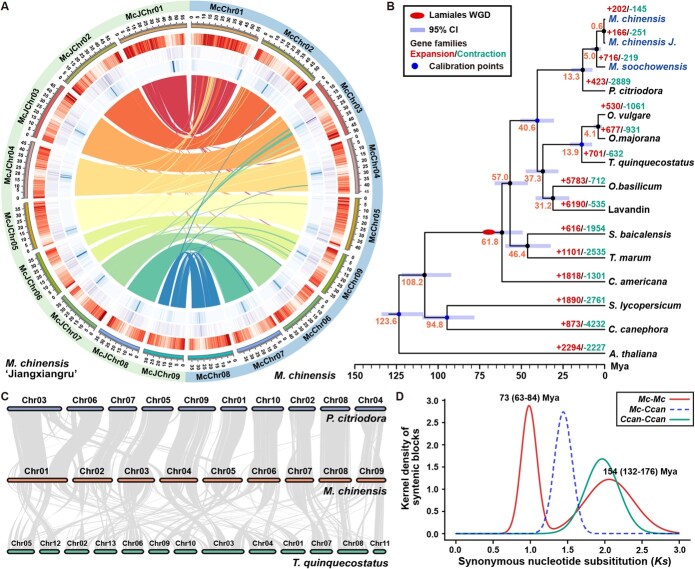
Assembly and evolutionary analysis of the *Mosla* genome. (A) Circos plot of *M. chinensis* and *M. chinensis* ‘Jiangxiangru’ genome. The chromosomal distribution of genes and the synteny within the genome are shown. Heatmap layers from the outside to the inside indicate chromosome name, gene density, and repeat elements across chromosomes (Copia and Gypsy, sequentially); inner lines represent syntenic blocks. (B) Phylogenetic tree based on single-copy genes, with divergence times estimated by MCMCtree. Gene family expansions and contractions were labeled on each tip. (C) Synteny analysis between the genomes of Mc, *P. citriodora*, and *T. quinquecostatus*. (D) *Ks* distribution of paralogous genes in *Mc–Mc*, Mc*–*Coffee, and Coffee*–*Coffee pairs. The peaks for common WGD of Lamiales, WGT of dicots, and divergence of Mc–Coffee are shown.

To provide a genetic basis for tracing and identifying the origin of Xiangru, the first chromosome-level genomes for Mc, McJ, and related Ms were generated. Based on K-mer distribution analysis (Fig. S3A–C), the estimated genome sizes for Mc, McJ, and Ms were 438.0, 410.6, and 385.0 Mb, respectively (Table 1, Table S4). Using PacBio HiFi sequencing (72–110× coverage) combined with Hi-C scaffolding (170×), we generated chromosome-level assemblies for Mc (426.1 Mb) and McJ (408.8 Mb), with 96.7%–97.2% of sequences anchored to nine pseudochromosomes (2*n* = 18), and a contig-level assembly for Ms (412.8 Mb). All assemblies showed excellent continuity and completeness, with the contig N50 values for all three genomes ranging from 38 to 44 Mb, and BUSCOs scores ranging from 98.2% to 98.7% (Fig. 2A, Table 1, and Fig. S3D and E).

Similar to other genomes of Lamiaceae plants, repeat sequences existed in *Mosla* genomes, which occupied 57.17%, 56.33%, and 55.83% of the Mc, McJ, and Ms genome sequences, respectively (Fig. 2A, Table S5). The majority of the repetitive sequences are retrotransposons, comprising 30%–32%, while another 18%–23% of the genome sequences are DNA transposons. Additionally, a very small number of rolling circles account for 0.04%–0.1% of the genome sequences. Based on the TE annotation, the long terminal repeat assembly index (LAI) was calculated for each genome, exhibiting 26.92 (Mc), 26.75 (McJ), and 18.44 (Ms) (Table 1), confirming gold-standard assembly quality. Gene prediction identified a total of 32 297, 33 245, and 34 133 protein-coding genes for Mc, McJ, and Ms, respectively, with BUSCO protein completeness scores ranging from 98.2% to 99.0%. In addition, telomeres were also annotated in Mc and McJ genomes, in which 11 telomeres were distributed on Mc genome and 12 telomeres on McJ genomes (Fig. S4).

Phylogenomic analysis of 15 dicots, including *Perilla citriodora*, *T. quinquecostatus, O. vulgare, Origanum majorana, Ocimum basilicum*, Lavandin (*Lavandula* × intermedia) from the Nepetoideae subfamily, *Teucrium marum, Scutellaria baicalensis*, and *Callicarpa americana* from the Lamiaceae, *Coffea canephora* and *S. lycopersicum* from the lamiids, as well as the model plant *Arabidopsis thaliana* from the superrosids as an outgroup, was conducted [27]. A total of 477 786 proteins (92.7%) from these 15 species were clustered into 33 504 orthogroups (Table S6). Mc, McJ, and Ms shared 16 692 orthologous gene families, with each containing around 225–294 unique orthogroups (Fig. S5A). As shown in Fig. 2B, we constructed a species phylogenetic tree using 417 (nearly) single-copy orthologous genes and analyzed the divergence times of the tree based on fossil evidence and previously reported divergence times. Divergence time estimation traced the split between Elsholtzieae and Mentha tribes to ~40.6 Mya (Middle Eocene), with *Mosla* radiating ~13.3 Mya (Miocene), contemporaneous with *Thymus* and *Origanum* diversification (13.9 Mya). Ms diverged from the branch of Mc and McJ about 5 Mya, while the divergence between Mc and McJ occurred around 0.6 (0.24–1.0, 95% highest posterior density) Mya, yet exhibited substantial structural variation on Chr04, Chr06, and Chr08 (Fig. S3F). Gene family dynamics revealed limited expansion (166 families) versus pronounced contraction (251 families) in McJ, supporting its origin from natural *Mosla* populations. As shown in Fig. 2C, exhibiting close genetic relationship with *P. citriodora*, Mc showed good collinearity with *Perilla*, while smaller block collinearity was obtained between Mc and *T. quinquecostatus*, which is consistent with divergence times.

**Figure 3:**
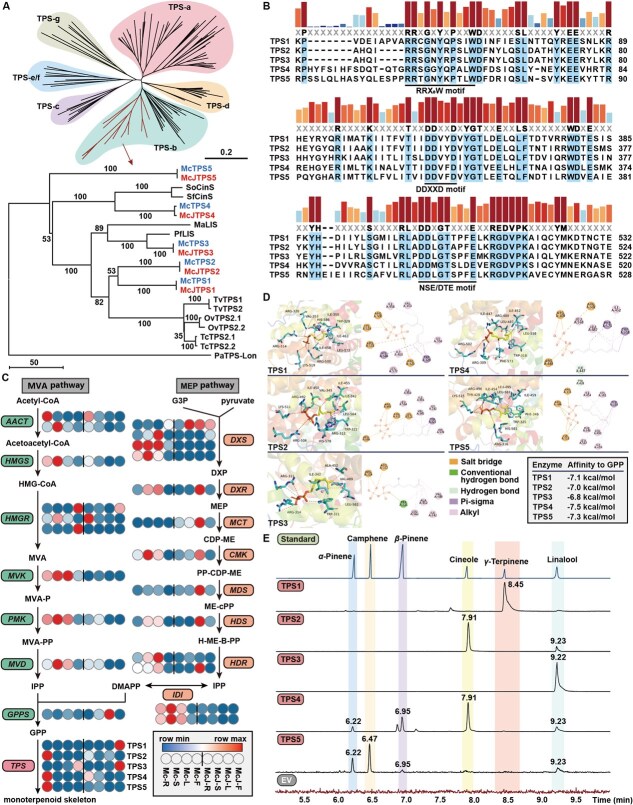
Functional characterization of TPS1–5. (A) Phylogenetic tree construction of TPS1–5. Amino acid sequences of TPSs are listed in Table S7. (B) Multiple sequence alignment of TPS1–5. (C) Expression of genes participating in monoterpenoid biosynthesis in different tissues of Mc and McJ. (D) Protein structures and molecular docking of TPS1–5 with GPP. (E) *In vitro* assay of TPS1–5 catalyzing GPP

Whole-genome duplication analysis identified two *Ks* peaks in Mc, a core eudicot γ-WGT event (~154 Mya) and a Lamiales-specific WGD (~73 Mya) (Fig. 2D), and McJ had a similar result as the Mc (Fig. S5B–E). In contrast with the whole-genome duplication patterns observed in other Lamiaceae lineages, recent paralog peaks reflected transposable elements (TE)-mediated local duplications (Fig. S5F), suggesting this mechanism drove gene family diversification in *Mosla*.

### Diversified monoterpenoids biosynthesis in *Mosla* mediated by TPS1–5

Metabolomic analysis identified diverse monoterpenoids including γ-terpinene in Xiangru, but their biosynthetic mechanism is still unclear. Phylogenetic analysis revealed five TPS-b family genes (TPS1–5) in both Mc and McJ, sharing extremely high sequence similarity (Table S7 and Fig. 3A). Notably, TPS4 clustered closely with cineole synthases from *Salvia* species, while TPS3 showed homology to linalool synthases from *Mentha aquatica* (Ma) and *Perilla frutescens* (Pf) (Fig. 3A). All TPS proteins contained conserved RRX_8_W motif, DDXXD motif, and NSE/DTE motif, critical for enzymatic activity, with DDXXD coordinating Mg^2+^ ions during catalysis (Fig. 3B).

RNA-seq was then performed on root, stem, leaf, and flower of Mc as well as McJ (Fig. S6A and B). For Mc, a total of 2144 differentially expressed genes (DEG) were found in the intersection of significantly DEG compared between leaf, stem, and flower with root samples, in which 896 were up-regulated, and 1248 were down-regulated (Fig. S6C). The results for McJ are similar to those for Mc, with 1713 genes up-regulated and 1729 down-regulated in the intersection of DEGs compared between leaf, stem, and flower with root samples (Fig. S6D). The expression level of DEGs of different tissues in Mc and McJ was given in Fig. S6E and F. Tissue-specific expression patterns of MVA/MEP pathway genes were concluded. While *DXR*, *MCT*, *CMK*, *MDS*, *HDS*, and *HDR2* in the MEP pathway showed higher expression in McJ leaves, rate-limiting enzymes (*DXS2/3/4*, *HDR2*, *IDI*) were more abundant in Mc. However, expression of *TPS1–5* showed no significant differences between species despite their distinct monoterpenoid profiles (Fig. 3C).

Molecular docking predicted strong binding affinities (−7.5 to −6.8 kcal/mol) between GPP and all five TPSs (Fig. 3D). Structural analysis revealed conserved interactions, that is, R residues forming salt bridges with GPP’s phosphate group, while hydrophobic residues (I, V, L) stabilizing the hydrocarbon chain. Unique features included hydrogen bonds in TPS4 (I447) and TPS5 (Y428, R496, K515), potentially enhancing catalytic efficiency.

**Figure 4:**
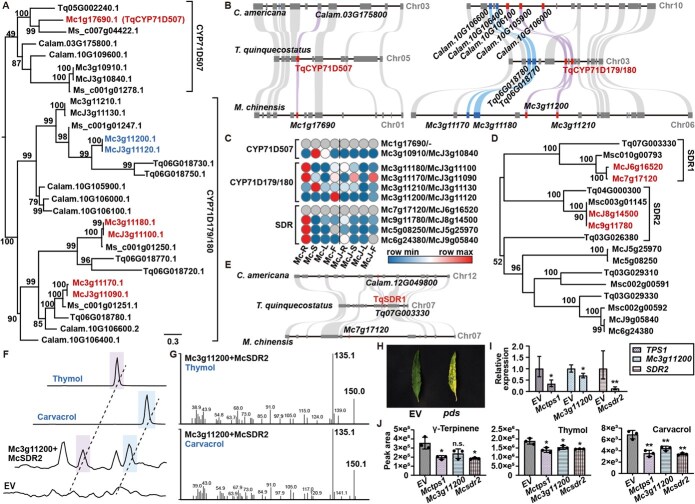
Evolution and biosynthesis of volatile monoterpenoid in *Mosla*. (A) Phylogenetic tree construction of CYP71Ds. (B) Collinearity analysis of CYP71D507 and CYP71D179 orthologs from *C. americana*, *T. quinquecostatus*, and Mc. (C) Expression of CYP71Ds and SDRs in Mc and McJ. (D) Phylogenetic tree construction of SDRs. (E) Collinearity analysis of SDR1 orthologs from *C. americana*, *T. quinquecostatus*, and Mc. (F) Transient transformation of Mc3g11200 in *N. benthamiana*. (G) Mass spectrometry of the product catalyzed by Mc3g11120 and McSDR2. (H) Feasibility verification of the VIGS system using *PDS*. (I) Expression of *McTPS1*, *McCYP71D179*, and *McSDR2* in *EV*, *Mctps1*, *Mc3g11120*, and *Mcsdr2* groups. (J) Relative content of monoterpenoids in *EV*, *Mctps1*, *Mc3g11120*, and *Mcsdr2* groups.

To examine the functions of TPS1–5, we performed enzymatic reaction *in vitro*, using GPP as substrate. As shown in Fig. 3E, results revealed that TPS1, consistent with the standard substance, exhibiting catalytic activities, resulted in the formation of γ-terpinene (Fig. S7A), which is the precursor of active metabolites thymol and carvacrol. The product of both TPS2 and TPS4 at 7.91 min was confirmed to be cineole (Fig. S7B). For TPS3, the catalytic product was found to peak at 9.22 min, which was consistent with linalool. Additionally, although the product of TPS2, TPS4, and TPS5 also exhibited close peaks at 9.23 min, the secondary mass spectrometry of these close peaks did not match the standard substance, losing characteristic ion peak at m/z 93 (Fig. S7C). TPS4 and TPS5 produced α-pinene and β-pinene (Fig. 4D and Fig. S7D and E), while TPS5 additionally yielded camphene (Fig. S7F). These findings establish TPS1 as the key enzyme initiating the carvacrol/thymol pathway via γ-terpinene synthesis, mirroring mechanisms reported in *T. vulgaris* [5] and *O. vulgare* [3]*.* The functional diversification of TPS2–5 explains *Mosla*’s capacity to produce varied monoterpenoid skeletons, providing a biochemical basis for its medicinal properties.

### Evolution and complete biosynthesis of volatile monoterpenoid in *Mosla* genus

As TPS1–5, clustered in one orthogroup, play an important role in the synthesis of diversified monoterpene skeletons, a more detailed phylogenetic analysis was conducted, revealing TPS1–3 cluster with TqTPS2 from *Thymus*, with TPS1/2 sharing a common ancestor (Fig. S8A). Synteny analysis showed TPS1 in Mc and McJ occupies a distinct genomic locus from TqTPS2 (Fig. S8B and C), suggesting transposon-mediated duplication. Notably, Mc possessed an additional TPS1 copy generated from recent tandem duplication, potentially explaining its elevated carvacrol and thymol content.

For downstream biosynthesis of carvacrol and thymol, CYP71Ds and SDR in Mc and McJ were analyzed, as CYP71D179/180, followed by SDR1, dominate accumulation of carvacrol and thymol in *T. vulgaris*. Based on the phylogenetic tree constructed with known function CYP71Ds, 18 orthologs of CYP71D were identified in Mc and are predicted to be functionalized in the oxidation of different types of terpene substrates (Fig. S9A). Evolutionary analysis revealed conserved tandem duplication of CYP71D179/180 clade genes in Lamiaceae (Fig. 4A and B, Fig. S9B). Several genes such as Mc3g11200, Mc3g11210, Mc3g11170, Mc3g11180 homologous with CYP71D179 highly expressed in the aerial part of Xiangru (Fig. 4C), and were picked out for the catalyzation of γ-terpinene. For the subsequent step, Mc7g17120/McJ6g16520 and Mc9g11780/McJ8g14500 were screened homologous with TqSDR1, on account of the orthologous gene grouping and collinearity analysis (Fig. 4D and E, Fig. S8C), but only Mc9g11780/McJ8g14500 was selected for functional verification, as RNA-seq revealed that both Mc7g17120 and McJ6g16520 were silent in all tested tissues of Xiangru (Fig. 4C).

To determine whether CYP71Ds and SDR in Xiangru were involved in the production of carvacrol and thymol, molecular docking between 4 CYP71Ds and γ-terpinene was performed, demonstrating a strongest binding affinity obtained in Mc3g11200 (Fig. S10). Transient transformation in *Nicotiana benthamiana*, a species that does not naturally produce phenolic monoterpenoids [5], was carried out. As homologous genes in Mc and McJ shared extreme similar amino acid sequences, functions of Mc3g11200 and SDRs in Mc were verified in volatile monoterpenoids production. As shown in Fig. 4F and G, leaves expressing Mc3g11200 and McSDR2 accumulated carvacrol and thymol; mass spectrogram also confirmed the production, indicating Mc3g11200 functions as both carvacrol and thymol synthase in volatile monoterpenoid biosynthesis of *Mosla*. Virus-induced gene silencing (VIGS) was employed to investigate the functional roles of *McTPS1*, *Mc3g11200*, and *McSDR2* in Mc. The VIGS system’s efficacy was validated through successful silencing of *PHYTOENE DESATURASE* (PDS), as evidenced by chlorophyll-deficient phenotypes (Fig. 4H). Upon introduction of VIGS vectors targeting *McTPS1*, *Mc3g11200*, and *McSDR2* into Mc, quantitative real-time polymerase chain reaction (qRT-PCR) analysis confirmed significant suppression of target gene expression (Fig. 4I). Subsequent metabolic profiling revealed accumulation of thymol and carvacrol drastically reduced in all three silenced lines (*Mctps1*, *Mc3g11200*, and *Mcsdr2*), while γ-terpinene biosynthesis prominently inhibited in *Mctps1* and *Mcsdr2* lines, indicating these three genes play important roles in functional monoterpenoid biosynthesis in *Mosla*, and furthermore establish complete carvacrol biosynthesis pathway, that is, TPS1 generates γ-terpinene as backbone; CYP71D179 (Mc3g11200) and SDR2 subsequently produce thymol and carvacrol.

### MIXTA, Woolly, and WIN1 co-regulate trichome development and monoterpenoid biosynthesis

**Figure 5:**
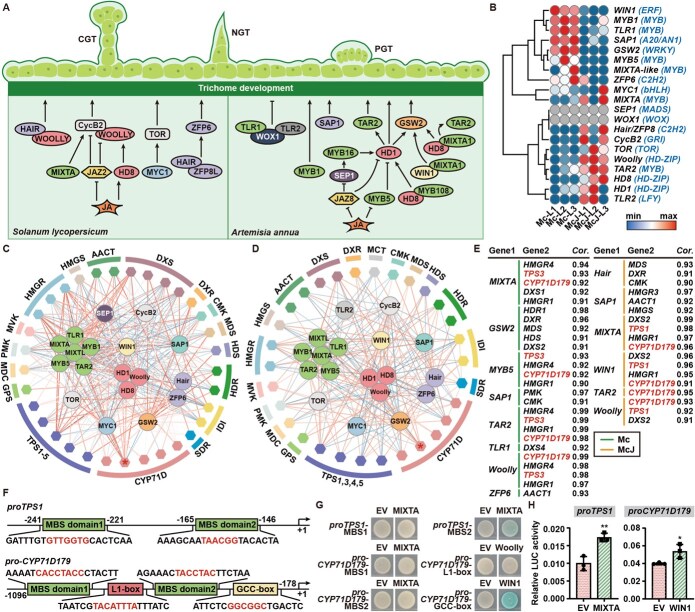
Correlation between trichome development and monoterpenoid accumulation in Mc and McJ. (A) Genetic network contributing to trichome development in *S. lycopersicum* and *A. annua*. (B) Relative expression of genes related to trichome development in leaves of Xiangru. (C, D) Co-expression analysis of genes participated in trichome development and monoterpenoid biosynthesis in Mc (C) and McJ (D). Gene pairs obtaining correlation coefficient >0.5 were exhibited. The thickness of lines indicated the strength of correlation. (E) Correlation coefficients of genes among genes related to trichome development and monoterpenoid accumulation. Gene pairs obtaining correlation coefficient >0.9 and *P* < 0.05 were exhibited. (F) Binding sites on *proTPS1* and *proCYP71D179*. (G) Y1H assay between MIXTA, Woolly, WIN1, and *proTPS1*, *proCYP71D179*. (H) Dual-LUC between MIXTA, WIN1, and *proTPS1*, *proCYP71D179*. Student’s *t*-test was performed for comparison, ^*^P < 0.05, ^**^P < 0.01.

As discrepant trichome densities observed between Mc and McJ, while trichome, especially CGT and PGT, have an important place for the biosynthesis and accumulation of volatile metabolites, the correlation between trichome development and monoterpenoid accumulation was subsequently analyzed. As shown in Fig. 5A, genes involved in trichome development in *S. lycopersicum* and *A. annua* were summarized. In leaves of Xiangru, the expression of genes related to trichome development exhibited significant species diversity (Fig. 5B). Transcription levels of *McGSW2* (homologous with *AaGSW2* [19]) and *McSAP1* (homologous with *AaSAP1* [23]) that directly regulate trichome density in Mc were 15.3- and 6.5-fold higher than that in McJ. In contrast, *McJHair* (homologous with *SlHair* [10]), which indirectly mediates trichome development by activating *ZFP6*, expressed 7.0-fold higher than that in Mc, while expression of *McJCycB2* (homologous with *SlCycB2* [28]) was 3.9-fold higher compared to Mc. The differential expression of genes directly or indirectly mediating trichome development might lead to the difference of trichome density between Mc and McJ. Additionally, *SEP1* and *WOX1* were not expressed in all tissues of Mc and McJ (Fig. 5B), indicating that no central function these 2 genes obtained in trichome development of Xiangru.

Co-expression between genes related to trichome development and monoterpenoid biosynthesis was also performed in Mc and McJ (Fig. 5C and D), indicating significantly high correlation between genes involved in secretory trichome development and monoterpenoid accumulation. Interestingly, as shown in Fig. 5E, genes participating in trichome formation such as *MIXTA*, *MYB5*, *TAR2*, and *Woolly* showed high correlation with *McTPS3* (linalool synthase), while *MIXTA*, *WIN1*, and *Woolly* exhibited a high correlation with *McJTPS1* (γ-terpinene synthase). In addition, the expression pattern of *CYP71D179* in both Mc and McJ showed extreme similarity with genes mediating trichome development.

As TFs account for the majority among genes reported to be associated with trichome development, it is interesting to explore whether certain TFs exist that mediate both trichome development and monoterpenoid biosynthesis in Xiangru. Promoters of *TPS1–5*, *CYP71D179*, and *SDR2* from Mc and McJ were then extracted from the genome to analyze *cis*-acting elements in the promoters. Conserved *cis*-acting elements such as binding sites of MYB, HD-ZIP, WRKY, ERF, C2H2, and bHLH were distributed on the promoters of Mc and McJ (Fig. S11), with *proTPS1* containing MBS domains and *proCYP71D179* harboring MBS domain, L1-box and GCC-box. As MIXTA (MYB), Woolly (HD-ZIP), and WIN1 (ERF) have similar expression pattern with *TPS1* and *CYP71D179*, interaction between MIXTA/Woolly/WIN1 and *TPS1*/*CYP71D179* were studied hereafter.

Since the binding sites sequences and distribution on the *TPS1* and *CYP71D179* promoters, as well as amino acid sequence of the three TFs were unanimous in Mc and McJ, we then selected *proMcTPS1*, *proMcCYP71D179*, McMIXTA, McWoolly, and McWIN1 represented in subsequent verification. A yeast one-hybrid (Y1H) assay was conducted to verify whether the three TFs target the promoters of *McTPS1* and *McCYP71D179 in vitro*. After introducing pB42AD-McMIXTA, pB42AD-McWoolly, and pB42AD-McWIN1 into EGY48A yeast strains containing potential binding domains of the target promoters, we found that strains integrating McMIXTA with *McTPS1*-MBS2 and WIN1 with *McCYP71D179*-GCC-box were able to grow on SD-Trp/-Ura plates and displayed blue color on SD-Trp/-Ura + X-α-gal plates (Fig. 5G). Dual-LUC assay was also conducted to verify the interaction *in vivo*. As shown in Fig. 5H, the luminescent activity of *proMcTPS::LUC* was elevated after introducing McMIXTA, resulting in a 1.75-fold increase compared with an empty vector, while McWIN1 increased the LUC activity of *proMcCYP71D179* by 1.35-fold. These results proved that *TPS1* is up-regulated by MIXTA, while WIN1 also positively regulates transcription of *CYP71D179*.

## Discussion

In the current naming system, McJ has two scientific names, in which one considered McJ as a natural variety, while the other one referred McJ as cultivar of Mc, while the latter is more widely recognized by the public. However, results of system evolution analysis and divergence time estimation underwent natural differentiation between Mc and McJ around 0.6 Mya ago. Genomic evidence further supports that McJ is initially a naturally formed resource rather than a variety developed through cultivation and domestication, and the impact of the artificial cultivation and selection process on the McJ population requires further population genetics research.

Parallel evolution is a kind of independent evolution that emerges in distantly related species, while the plant specialized metabolites are important traits in studies of evolutionary trajectories, like cannabinoid synthesis catalyzed by homologous enzymes in *Helichrysum umbraculigerum* and *Cannabis sativa* [29]. In our study, we infer that the transposon-mediated duplication led to the formation of γ-terpinene synthase with neofunctionalization in Mc and McJ, whereas in *T. quinquecostatus* (Fig. S8B), γ-terpinene synthase was generated through local tandem duplication and functional divergence [6]. TPS1 from Mc/McJ and TqTPS2 have a common origin and demonstrate parallel evolution to achieve similar enzyme functions (Fig. S8A). Additionally, it is notable that γ-terpinene synthase (TPS1) was absent in *P. citriodora* (Fig. 2B and Fig. S7A), which is consistent with its lack of carvacrol and γ-terpinene production [30] in *P. frutescens* (a cultivated species and autotetraploid of *P. citriodora*) [31].

**Figure 6:**
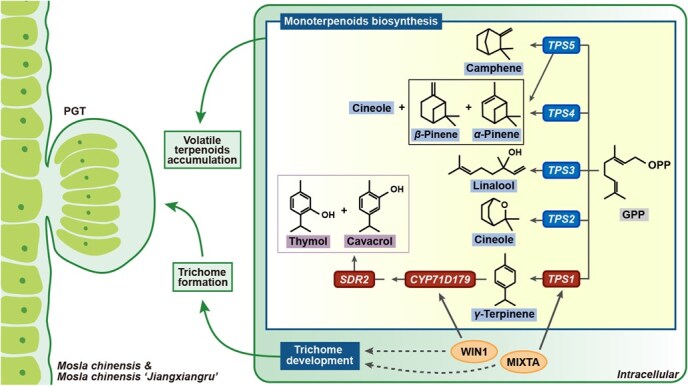
Molecular mechanism for quality formation in Xiangru

Trichomes represent specialized epidermal structures that have evolved in plants as adaptive features against biotic and abiotic stresses [32]. As a wild species, Mc predominantly inhabits grasslands and forest understories in Jiangxi, where natural selection pressures likely favor higher trichome density to enhance stress resilience. In contrast, McJ has undergone domestication and cultivation, reducing exposure to environmental stressors and potentially leading to relaxed selection on trichome development. This is consistent with our observation that Mc exhibits significantly greater trichome density and associated monoterpenoid accumulation compared to McJ. Trichomes serve as storage sites for a diverse array of secondary metabolites, including terpenoids, phenylpropanoids, and flavonoids [33]. Volatile terpenoids, such as artemisinin in *A. annua* [34], menthol in *Mentha arvensis* [35], and linalool in *Lavandula* genus [36], are particularly prominent examples of trichome-localized specialized metabolites. Our metabolomic data revealed that Mc accumulates higher levels of monoterpenoids, including linalool (catalyzed by TPS3), while McJ shows preferential accumulation of γ-terpinene in leaves and flowers (Fig. 1J). These findings underscore a strong correlation between trichome development and VOC biosynthesis in Xiangru. In addition, as a regulator of plant growth processes, phytohormone, such as auxin, jasmonates, gibberellin, plays important roles in trichome development, and genes related to phytohormone biosynthesis as well as signal transduction also contribute to trichome initiation and density [37–39]. Interestingly, it is found in our research that McJAZ8 (McJ2g02820) exhibited reduced expression in leaves (Fig. S12), which potentially promotes trichome development in McJ, due to the inhibited effect of JAZ in trichome formation.

TFs have emerged as central regulators linking morphological traits with specialized metabolism in medicinal plants. For instance, PgMADS41 and PgMADS44 co-regulate root architecture and ginsenoside biosynthesis in *Panax ginseng* [40], PnMYB31/PnMYB78-PnbHLH31 mediates both nail head formation and ginsenosides accumulation [41], and SmARF10/16/17 negatively regulates tanshinone biosynthesis and promotes hairy root growth [42]. In *S. lycopersicum*, SlWoolly integrates trichome density with terpenoid biosynthesis via regulation of *SlTPS* [43], while ARF1 accelerates both secretory trichome development and artemisinin production in *A. annua* [44]. Our study extends this paradigm to *Mosla*, demonstrating that MIXTA and WIN1, which are reportedly strongly associated with trichome development, directly modulate VOC accumulation by binding to promoters of *TPS1* and *CYP71D179*, offering novel targets for quality-directed breeding in Xiangru.

Through integrative analysis of morphological, genomic, and metabolic data, this study found differences in biomass, trichome density, and VOC content between Mc and McJ, in which higher content of thymol and carvacrol in Mc was obtained by generating additional copies of *TPS1*. Furthermore, this study also clarified the quality formation mechanism of Xiangru through *in vivo* and *in vitro* functional characterization. As summarized in Fig. 6, we identified five functionally diversified monoterpene synthases TPS1–5 responsible for producing γ-terpinene, cineole, linalool, α-pinene, β-pinene, and camphene, in which γ-terpinene transformed by TPS1 is further principally catalyzed to carvacrol and thymol via CYP71D179 and SDR2. We also established that the TFs MIXTA and WIN1 form a regulatory module that simultaneously governs trichome development and monoterpenoid biosynthesis, thereby shaping the characteristic medicinal quality of Xiangru. These findings fill the vacancy of *Mosla* genomes, provide fundamental genetic resources for investigating terpenoid metabolism in *Mosla*, and establish a framework for molecular-assisted breeding to enhance its pharmaceutical value.

## Materials and methods

### Plant materials

The Mc, McJ, and Ms cultivar were collected in Yiyang County, Jiangxi Province, China. For whole-genome sequencing, healthy and tender leaves were collected in June 2023, with the external contaminants removed. Meanwhile, the roots, stems, leaves, and flowers of McJ and Mc were collected for RNA-seq and metabolome detection. Plant tissues were carefully removed, immediately snap-frozen in liquid nitrogen, and stored at −80°C.

### Agronomic characters and peltate glandular trichome density analysis

The plant height, plant width, stem diameter, leaf length, and leaf width of 1-year Mc and McJ were measured to characterize the differences in their agronomic traits. Trichomes of 1-year Mc as well as McJ under fields at the same magnification (1.9 mm × 2.9 mm) were visualized using an Olympus microscope equipped with a DP72 digital imaging system and scanning electron microscope (S-4800; Hitachi, Tokyo, Japan). Image J software (https://imagej.nih.gov/ij/) was used to count PGTs and measure leaf area. The experiment consisted of 24 biological replicates.

### Compound quantification

Aerial tissues from McJ and Mc were subjected to lyophilization and subsequent mechanical homogenization. Precisely weighed 100-mg of the above tissues for cold methanol ultrasonic extraction for 1 h. The supernatant was collected through centrifugation at 13000 × *g* for 10 min and filtered through a 0.22-μm syringe filter prior to chromatographic analysis. GC–MS separation was performed on a TRACE 1310 GC system (Thermo Scientific) coupled with TSQ 8000 triple quadrupole MS employing helium carrier gas (1 mL/min constant flow) through a TG-5MS capillary column (30 m × 0.25 mm ID, 0.25-μm film) under splitless injection mode. The programmed temperature sequence was 50°C (3 min hold), ramp to 90°C by 15°C/min, then to 150°C by 5°C/min, and ramp to 300°C by 100°C/min (2-min hold). Compounds were identified by comparison with the NIST (National Institute of Standards and Technology) database library.

### Analysis of volatile organic compounds by headspace solid-phase microextraction

Determination of VOCs in roots, stems, leaves, and flowers of Mc and McJ was performed using headspace solid-phase microextraction, conducted by Shanghai Biotree Biotech [45]. Further data analysis including clustering, principal component analysis (PCA), and OPLS-DA was performed using SIMCA 14.0 software. Hierarchical clustering was performed using Morpheus online software.

### Genomic and transcriptomic analysis

Methods of genome assembly, annotation and comparative genomic analysis, as well as RNA-seq and analysis were provided in Method S1.

### Sequence alignment, phylogenetic analysis, and promoter element prediction

Multiple sequence alignments of the amino acid sequence were performed using Snapgene (v4.3.6). Phylogenetic analysis employing the neighbor-joining method in MEGA v.8.0 involved 1000 bootstrap replicates to evaluate tree topology support, then visualized using iTOL (https://itol.embl.de/). The sequences of terpenoid synthases used for constructing the phylogenetic tree were obtained from previous reports [46]. The promoter element prediction was performed using the online software New PLACE (https://www.dna.affrc.go.jp/PLACE/?action=newplace).

### Structure modeling and molecular docking

AlphaFold2 was used for modeling McJTPS1–5, allowing for energy optimization and adjustment of force field parameters. The chemical structure of GPP was obtained from the PubChem compound database (https://pubchem.ncbi.nlm.nih.gov/) and prepared using ChemDraw. Subsequent energy minimization of this structure was executed using the MM2 force field within the Chem3D program. For the molecular docking studies between the ligand (GPP) and the receptor proteins (McJTPS1–5), AutoDock Vina version 1.1.2 was employed, and the active site of each receptor was precisely defined using AutoDockTools. Conformations with high scores were selected and visualized using PyMOL v2.2.0 and Ligplus. Furthermore, the 2D graph was analyzed using Discovery Studio 2020 Client.

### *In vitro* enzymatic catalysis

The coding sequences of McTPS1–5 were directionally ligated into pMAL-C2x and introduced into Rosetta (DE3) chemically competent cells. Recombinant protein expression was induced under optimized conditions (0.5 mM isopropyl-β-D-thiogalactopyranoside (IPTG), 28°C, 12 h with 220 rpm orbital shaking). Purification of the target proteins was achieved using Amylose Resin High Flow (NEB, Ipswich, MA, USA). The purified protein was incubated for 3 h in a reaction mixture containing 50 μM geranyl pyrophosphate (CAS: 116057-55-7, Cayman), 10 mM MgCl_2_, 50 mM 4-hydroxyethylpiperazine ethane-sulfonic acid (HEPES) (pH 7.5), 5 mM dithiothreitol (DTT), and 5% glycerol. The reaction product was extracted with n-hexane and subsequently filtered using a 0.22-μm filter, and then detected using GC–MS. Primers are listed in Table S8.

### Transient expression in *N. benthamiana*

The full-length cDNA of *McCYP71Ds* (Mc3g11200, Mc3g11210, Mc3g11170, Mc3g11180, Mc3g10910) and *McSDR2* (Mc9g11780) was constructed into the pHER vector and transformed into GV3101 competent cell. Functional verification in *N. benthamiana* was performed as described previously [5].

### Virus-induced gene silencing

A 300-bp nonconservative domain of McTPS1, McCYP71D179, and McSDR2 was cloned into the pTRV2 vector, and the recombinant plasmids pTRV1 and pTRV2 were transformed into GV3101 (pSoup-p19) competent cell. Bacterial suspensions were adjusted to an OD_600_ of 0.9 using VIGS buffer. Subsequently, equal volumes of pTRV1 and pTRV2 (or pTRV2-gene) were mixed uniformly and then infiltrated into microwounded, young tender leaves of 3-month-old Mc plants. After rinsing with ddH₂O to eliminate *Agrobacterium*, plants underwent 24 h of darkness followed by 14 days of light exposure. New leaves were harvested for qRT-PCR analysis and monoterpene content determination. The efficacy of the VIGS system was validated using PDS as a control.

### Yeast one-hybrid assay

The full-length coding sequences of McMIXTA, McWoolly, and McWIN1 were cloned into the pB42AD vector, while three tandem repeats of *cis*-acting elements (MBS domain, L1-box, and GCC-box) predicted in the promoters of *McTPS1* and *McCYP71D179* were cloned into pLacZ vectors. Different combinations of pB42AD plasmids and pLacZ plasmids were co-transformed into yeast strain EGY48A and cultivated on SD-Trp/-Ura medium with X-α-gal. The empty vector pB42AD was used as negative controls. Primers are listed in Table S8.

### Dual-LUC luciferase assay

The dual-LUC luciferase assay was performed as described previously [47]. The full-length ORF of McMIXTA and McWIN1 was ligated into the pHB vector, while the promoter of *McTPS1* and *McCYP71D179* was cloned into the pGREENII0800 vector. The above engineered plasmids were transferred into *Agrobacterium tumefaciens* GV3101, and then co-infiltrated into leaves of *N. benthamiana*.

## Supplementary Material

Web_Material_uhaf263
