# Supplementary material for: Integrative chromosome-level genomics and metabolomics uncover regulatory networks linking monoterpenoid biosynthesis and glandular trichome formation in Mosla chinensis
Source: Hortic Res. 2025 Oct 1;13(1):uhaf263. doi: 10.1093/hr/uhaf263 (PMC12861481; doi:10.1093/hr/uhaf263)
Supplement: Web_Material_uhaf263 [file web_material_uhaf263.zip › Supplemental Figures.docx]

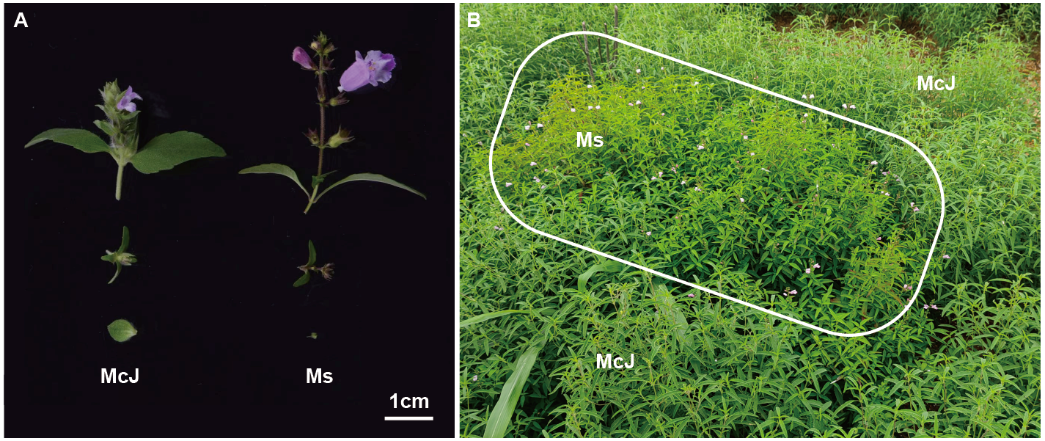


**Figure S1.** **Morphology and distribution of McJ and Ms**

(A) Flower characteristics of McJ and Ms. (B) Ms occasionally distributed in McJ plantation base.


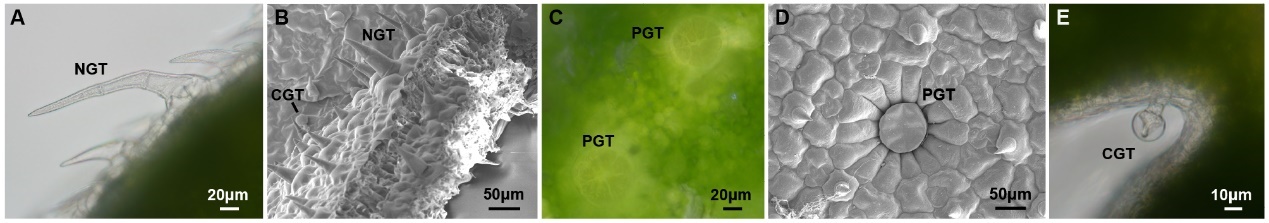


**Figure S2.** **Trichomes in Mc and McJ.**

(A-B) NGT on leaf surface. (C-D) PGT on leaf surface. (E) CGT on leaf surface.


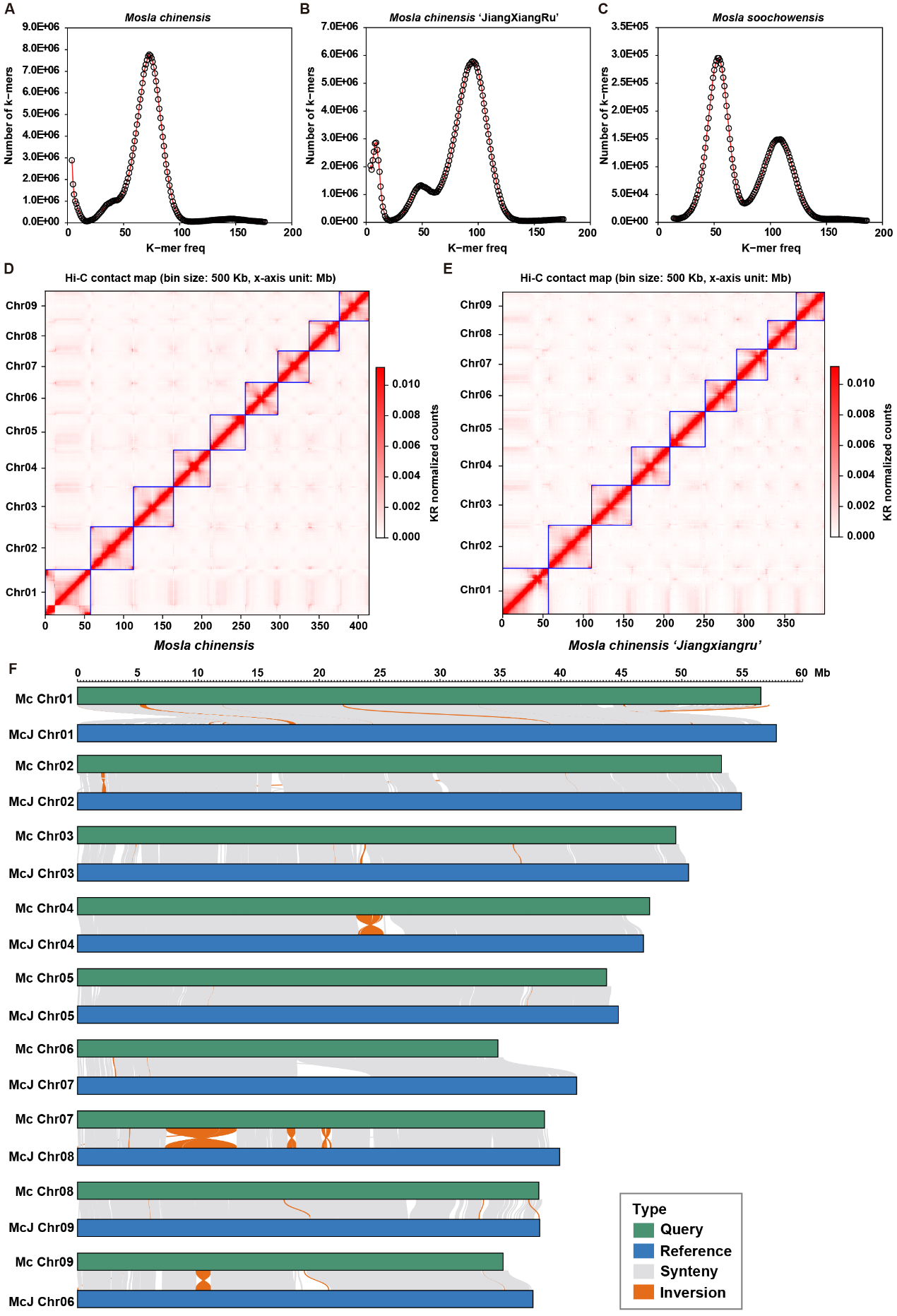


**Figure S3. Genomes of Mc, McJ and Ms**

(A-C) K-mer distribution of Mc, McJ and Ms sequencing reads. (D-E) Heatmap of chromosome anchoring using Hi-C for Mc and McJ. (F) Genome synteny between Mc and McJ based on MUMMER alignment of genomic sequences.


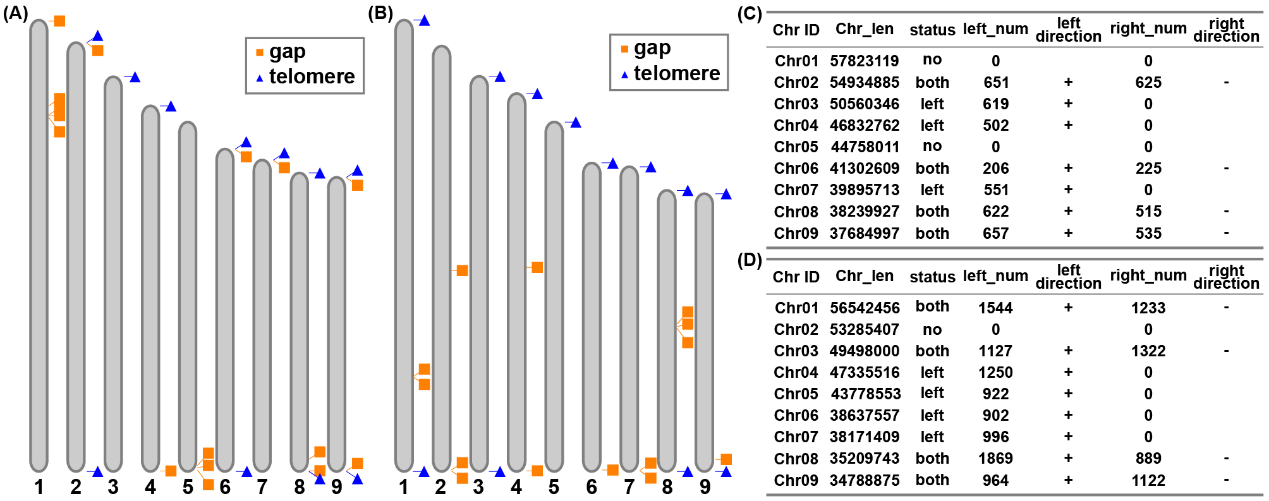


**Figure S4. Annotation of telomeres in Mc and McJ genomes**

(A) Distribution of telomeres in Mc genome. (B) Distribution of telomeres in McJ genome. (C) Detailed information of telomeres in Mc genome. (D) Detailed information of telomeres in McJ genome.


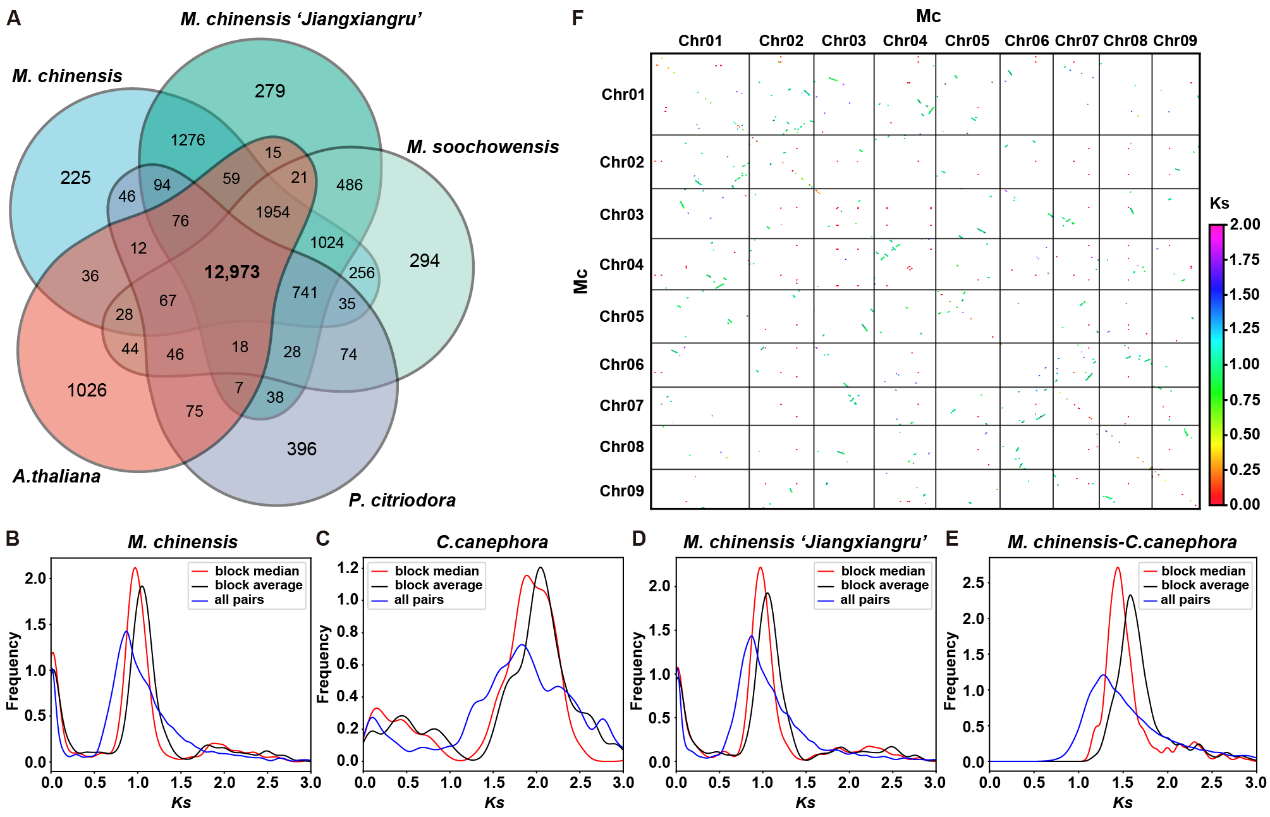


**Figure S5. Orthogroups and whole genome duplication**

(A) Venn diagram of orthologous families and genes. (B-E) Distrubution of *Ks* between paralogous genes in Mc, McJ, Ms and Coffee. (F) Syntenic dot plots show collinearity compare within Mc genome and *Ks* of synteny blocks.


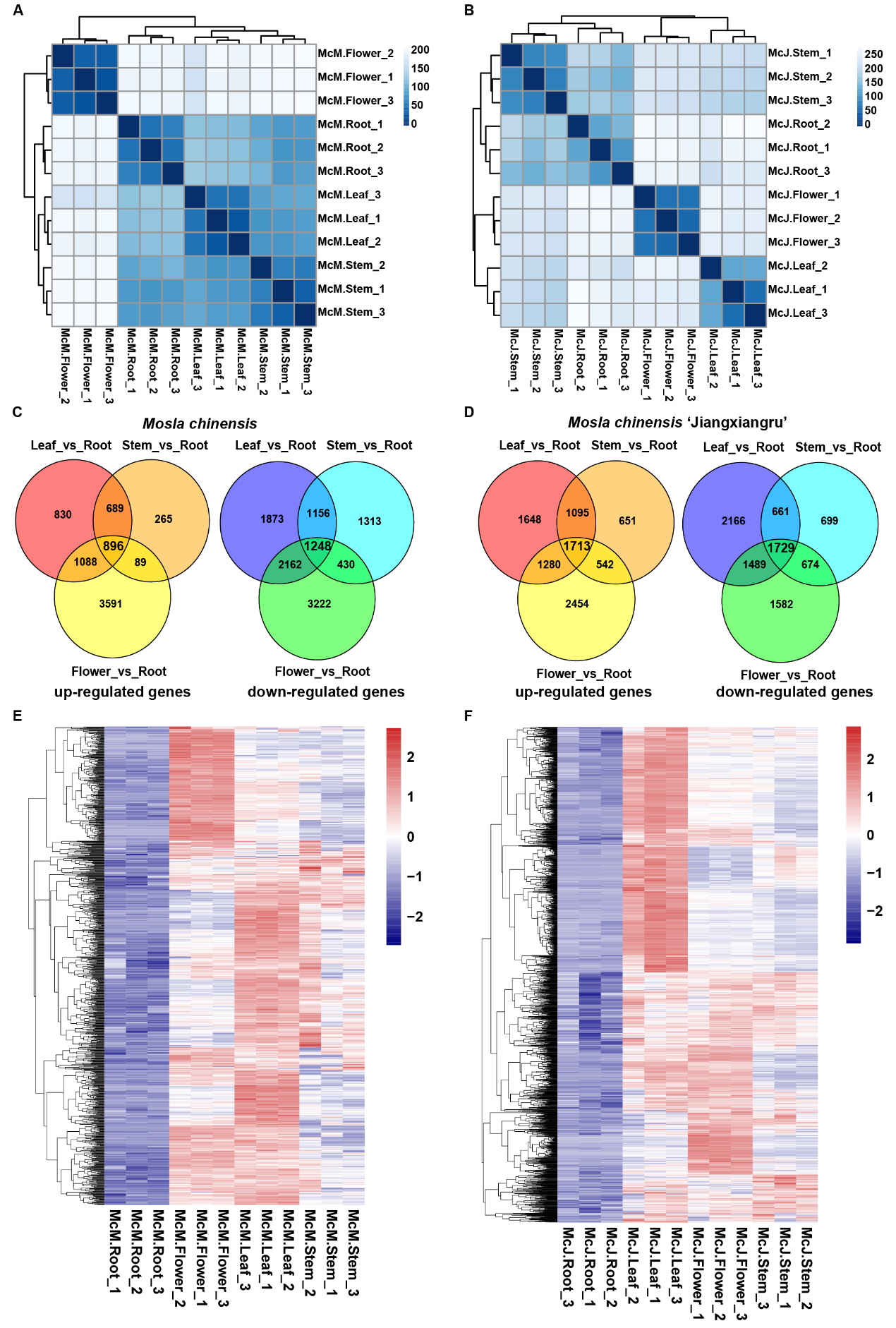
**Figure S6. RNA-Seq and differential gene expression analysis of Mc and McJ**

(A-B) Heatmap of gene expression level correlation of root, leaf, stem and flower samples in Mc and McJ.

(C-D) Venn diagram of up- or down-regulated genes for each DEG sets in Mc and McJ.

(E-F) Heatmap of up-regulated genes of Mc and McJ.


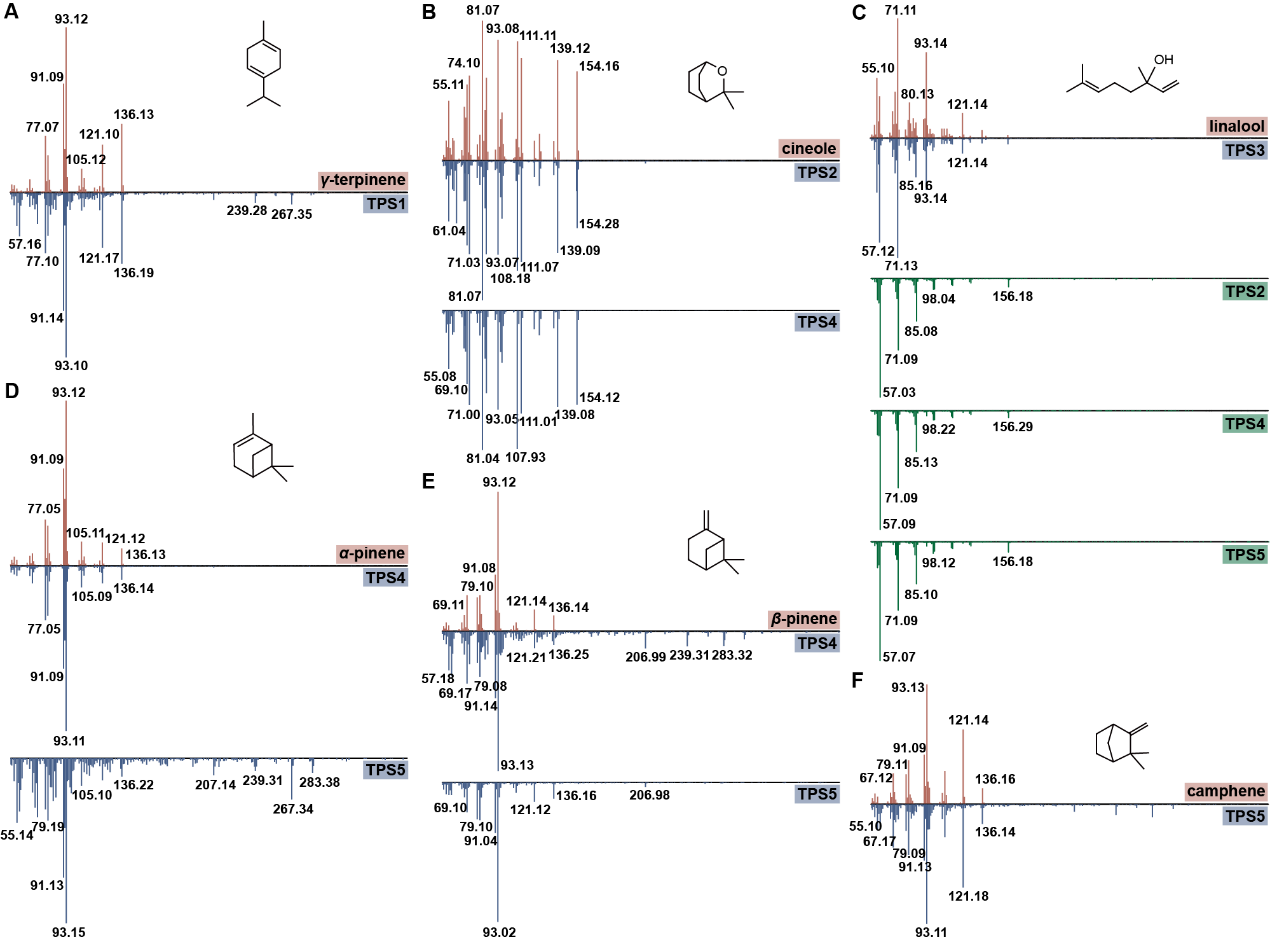


**Figure S7. Secondary mass spectrometry of standard substance and products of TPS1-5.**

(A) Secondary mass spectrometry of γ-terpinene and product of TPS1. (B) Secondary mass spectrometry of cineole and products of TPS2 and TPS4. (C) Secondary mass spectrometry of linalool and products of TPS2-5. (D) Secondary mass spectrometry of α-pinene and products of TPS4 and TPS5. (E) Secondary mass spectrometry of β-pinene and products of TPS4 and TPS5. (F) Secondary mass spectrometry of camphene and product of TPS5.


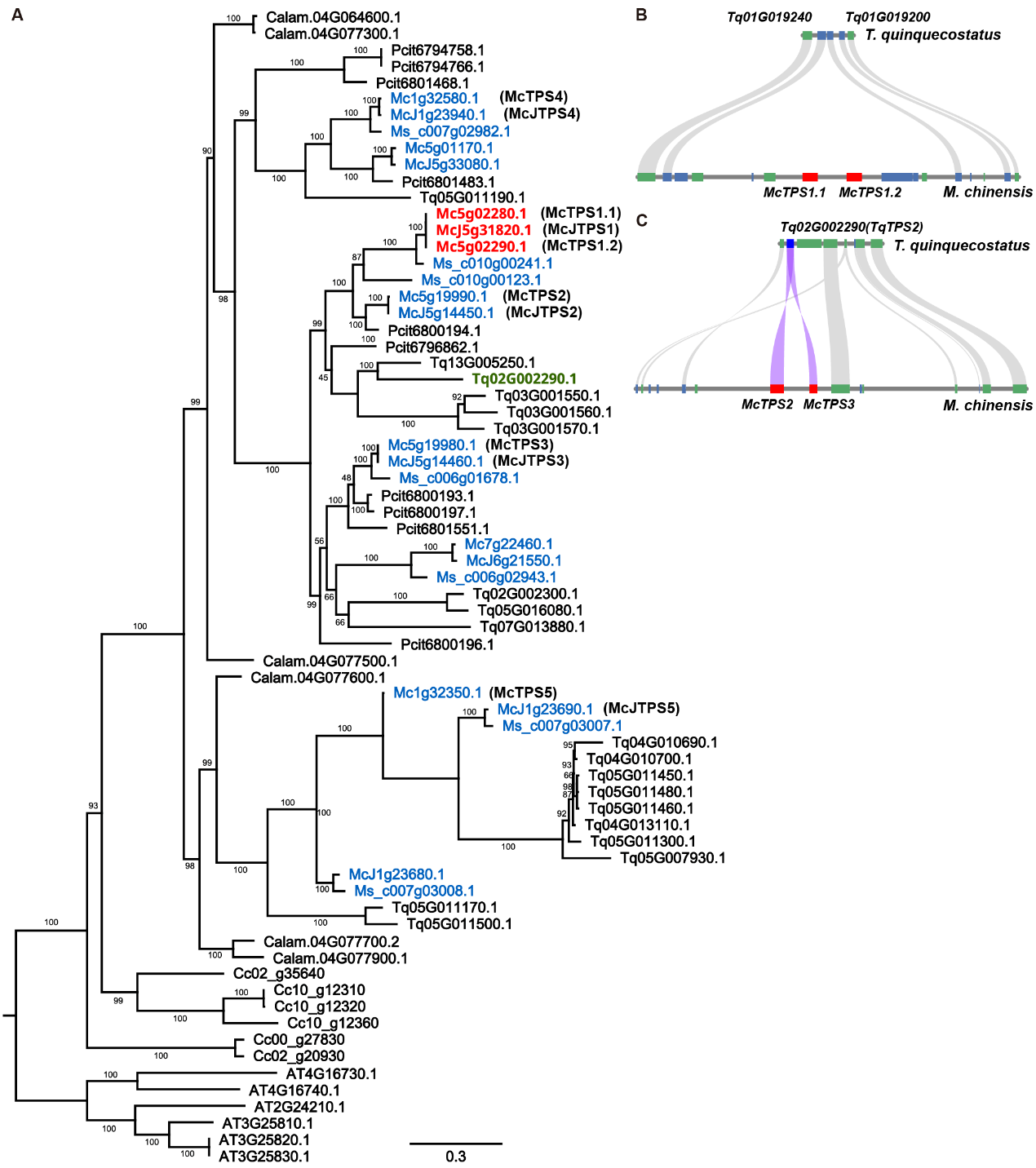


**Figure S8. Phylogenetic tree and micro-synteny of TPSs.**

(A) Phylogenetic tree constructed using maximum likelihood algorithms of TPS1-5 and its orthologs in related species. Node label showed the bootstrap values. (B-C) Micro-synteny of genomic regions in which *McTPS1-3* located between Mc and *T.quinquecostatus*.


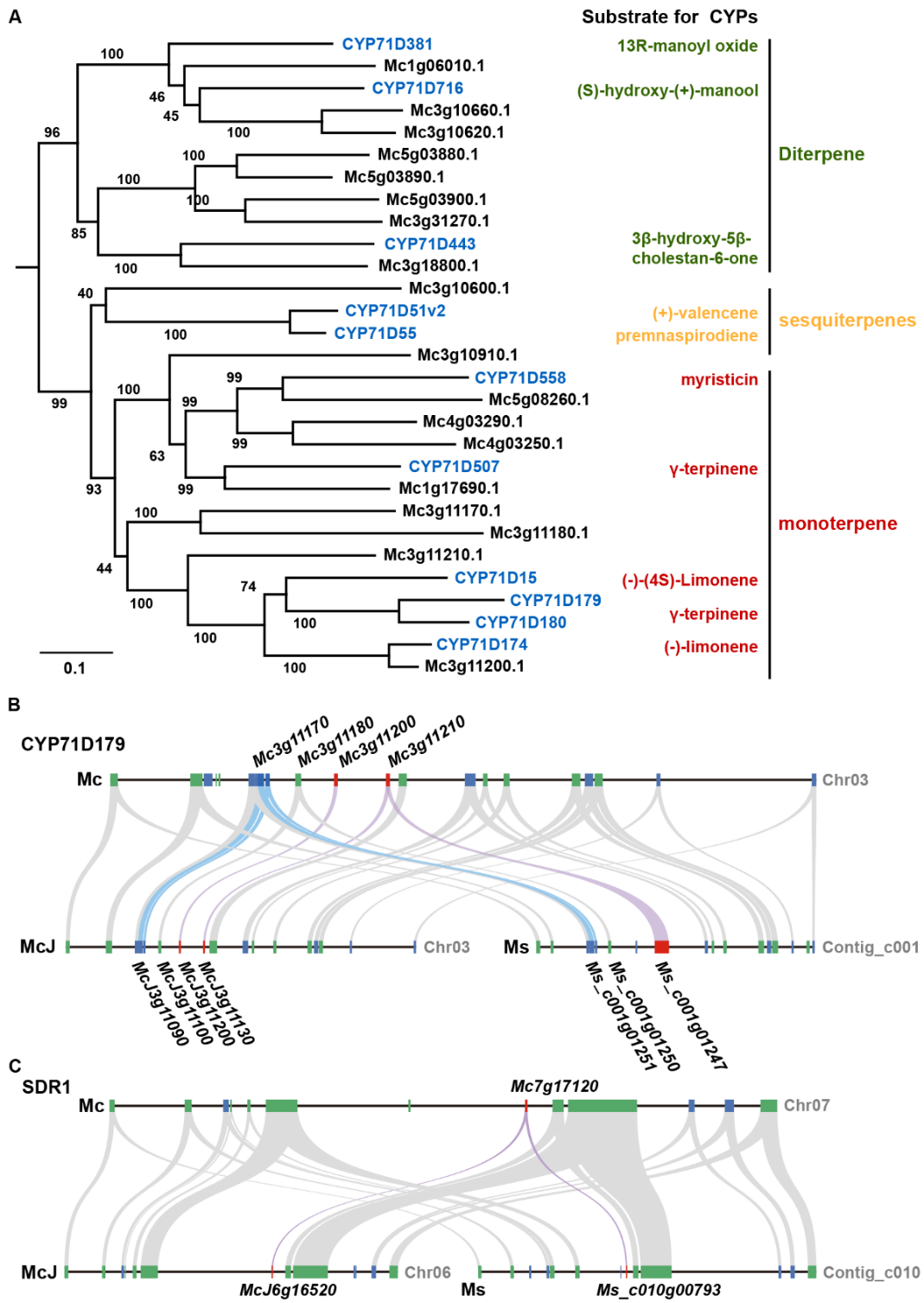


**Figure S9. Phylogenetic tree and micro-synteny of CYP71Ds and SDR.**

(A) Phylogenetic tree constructed using maximum likelihood algorithms of the CYP71Ds in Mc. Node label showed the bootstrap values. Right panel labeled the substrate for each characterized reference CYP71Ds. (B) Micro-synteny of tandem duplication region of CYP71D orthologs located among Mc, McJ and Ms. (C) Micro-synteny of SDR1 ortholog among Mc, McJ and Ms.


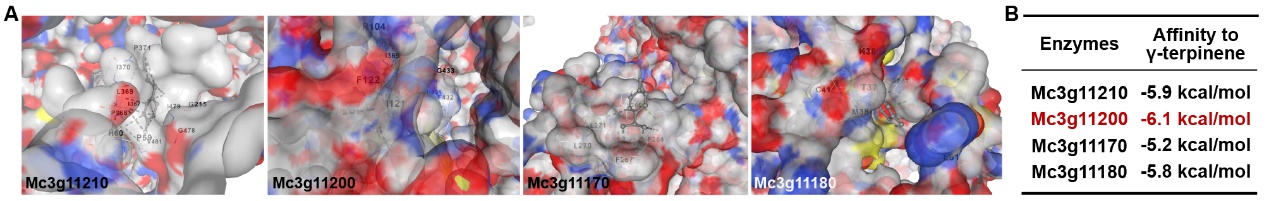


**Figure S10. Molecular docking of McCYP71Ds with γ-terpinene**

(A) Molecular docking models of McCYP71Ds with γ-terpinene. (B) Binding affinities of 4 McCYP71Ds with γ-terpinene.


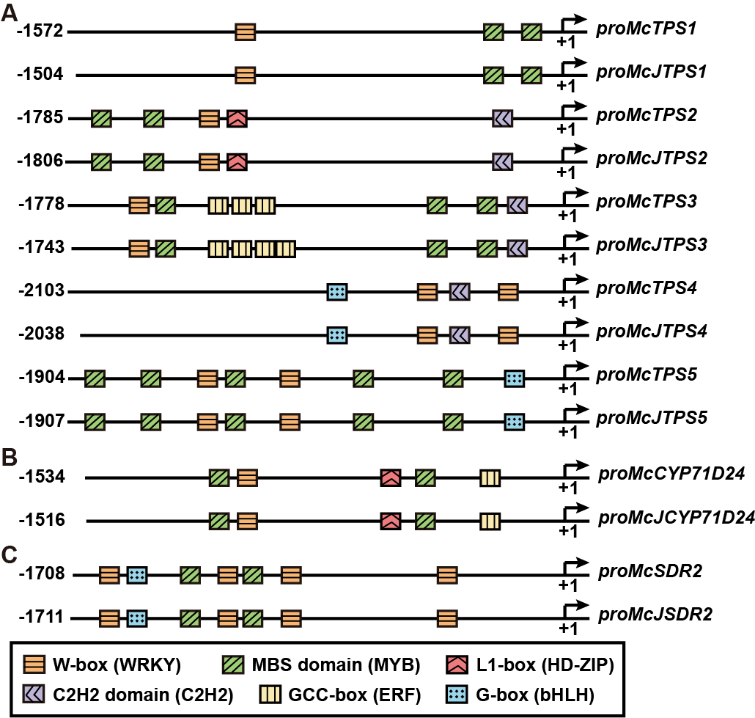


**Figure S11. *Cis*-elements on the promotor of monoterpenoid biosynthetic genes.**

(A) *Cis*-elements on *proMcTPS1-5* and *proMcJTPS1-5*. (B) *Cis*-elements on *proMcCYP71D179* and *proMcJCYP71D179*. (C) *Cis*-elements on *proMcSDR2* and *proMcJSDR2*.


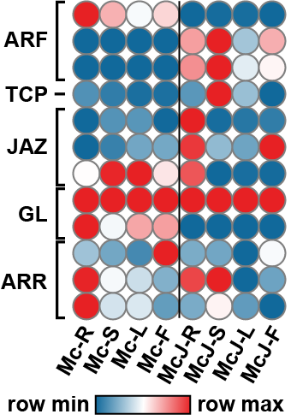


**Figure S12. Expression of genes related to phytohormone**
