# Supplementary material for: Integrative chromosome-level genomics and metabolomics uncover regulatory networks linking monoterpenoid biosynthesis and glandular trichome formation in Mosla chinensis
Source: Hortic Res. 2025 Oct 1;13(1):uhaf263. doi: 10.1093/hr/uhaf263 (PMC12861481; doi:10.1093/hr/uhaf263)
Supplement: Web_Material_uhaf263 [file web_material_uhaf263.zip › Supplemental Methods.docx]

# **SUPPLEMENTARY METHODS**

**Genome assembly and annotation**

The plants of Mc, McJ and Ms were collected from JiangXi province and identified by professor Yu Jinbao, then cultivated in a greenhouse of the Center for Chinese Materia Medica Resources, China Academy of Chinese Medical Sciences in Beijing. High-quality genomic DNA was extracted from the young leaves using Qiagen Genomic DNA kit. To obtain the HiFi reads, the PacBio libraries were prepared using the purified genomic DNA fragments and sequenced on a PacBio Revio platform. For chromosome anchoring, Hi-C libraries were constructed and sequenced on Illumina platform with 150 pair-end reads. Genome size was estimated by K-mer analysis and the HiFi reads were assembled using HiFiasm v0.21.0-r686 (Cheng et al., 2021) with default parameter, applied with the Hi-C reads to obtain the phased contig assembly. The Hi-C reads were trimmed and qualified with fastp (Chen et al., 2018), mapped to contigs with BWA, and used to anchor the contigs into chromosomes using HapHiC v1.0.6 (Zeng et al., 2024) software, all reads alignments were filtered with the parameters MAPQ 1 (mapping quality ≥ 1) and NM 3 (edit distance < 3). The contig were iteratively corrected for 5 times (--correct_nrounds 5). We kept the default settings for other parameters. To annotate the genome, total RNA was extracted from different organs using Qiagen Plant RNA kit. and performed pair-end sequencing on Illumina nova 6000 platform for each sample. Otherwise, Mixed cDNA library was constructed for Mc, McJ and Ms and generated full-length transcriptome sequencing on ONT platform.

Repeat annotation of the genomes was implemented using EDTA (The Extensive de novo TE Annotator, v2.2.0) pipeline (Ou et al., 2019). Gene structures were predicted based on the softmasked genome sequences using the Braker 3 (Gabriel et al., 2024) with the mapped RNA-seq data as evidence, which utilized AUGUSTUS v3.5.0 (Stanke et al., 2006) and GeneMark v3.68 for *ab initio* annotation. Additionally, gene structures were predicted by the Helixer v0.34.0 (Stiehler et al., 2020) and Transdecoder v5.7.1. For transcriptome-based annotation, RNA-seq data was assembled with StringTie v2.2.3. the protein sequences from SwissProt and protein sequences from Lamiaceae reference genomes were aligned to the genome using MiniProt v0.13-r248 (Li, 2023) for homology-based annotation. all annotations were integrated and validated with EvidenceModeler v2.1.0 (Haas et al., 2008). The protein sequences of protein coding genes were analyzed with InterProScan (Jones et al., 2014), EggNOG mapper (Cantalapiedra et al., 2021; Huerta-Cepas et al., 2019) and combined with BLASTP searches (against the Nr, SwissProt and AraProt11 database) for functional and GO annotations. Transcription factors were identified using the online database PlantTFdb (Jin et al., 2017). We used Blastp to compare the protein sequences with the database of Nr, SwissProt and Araprot11 (Cheng et al., 2017). Genome and protein-coding genes completeness was assessed with BUSCO v5.7.0 (Manni et al., 2021), using green plants datasets (embryophyta_odb10). and the LAI values were calculated with EDTA pipeline.

**Comparative genomic analysis**

Orthologous gene families of the selected species ware obtained with OrthoFinder v2.5.5. To reconstruct the phylogenetic tree of and related species, Protein sequences of single-copy orthologous genes were aligned using MAFFT (Katoh et al., 2002), and the alignment was converted to nucleotide with PAL2NAL (Suyama et al., 2006) and trimmed using TrimAL (Capella-Gutiérrez et al., 2009). Subsequently, the nucleotide matrix was concatenated and 4dTV sites were extracted using MEGA 10 (Tamura et al., 2021). For maxima-likelyhood tree construction, the GTR+G4 model was selected using ModelTest-NG (Darriba et al., 2020), and phylogenetic tree construction was performed using RAxML-NG (Kozlov et al., 2019), with 2000 bootstraps. divergence time of species was analyzed using MCMCtree of PAML suite (Yang, 2007), the fossil calibration times was obtained from the literature. We used CAFE5 (Mendes et al., 2020) to analyze the expansion and contraction of gene families. Phylogenetic tree was visualized using ggtree. Gene collinearity and duplication were analyzed with MCScanX-transposed (Wang et al., 2013). Whole-genome duplication analysis was performed using the WGDI v0.74 (Sun et al., 2021), which employed ParaAT for Ka/Ks calculation. Visualization was conducted using the CICROS (Krzywinski et al., 2009) and JCVI (Tang et al., 2024).

*Perilla citriodora* (Zhang et al., 2021), *T. quinquecostatus* (Sun et al., 2022), *O. vulgare, O. majorana* (Nolan et al., 2020)*,* *Ocimum basilicum* (Gonda et al., 2020), Lavandin (*Lavandula* × intermedia) (Li et al., 2023) from the Nepetoideae subfamily, *Teucrium marum* (Smit et al., 2024)*, Scutellaria baicalensis* (Pei et al., 2023) and *Callicarpa americana* (Hamilton et al., 2020) from the Lamiaceae, *Coffea canephora* (Salojärvi et al., 2024) and *S. lycopersicum* (Consortium, 2012) from the lamiids, as well as the model plant *Arabidopsis thaliana* (Cheng et al., 2017) from the superrosids were selected to conduct phylogenomic analysis.

**RNA-seq and Analysis**

RNA-seq data were generated form the root, stem, leaf and flower tissues of three *Mosla* species. Each group contained three biological replications. mRNA was used to construct the RNA-seq libraries and sequenced on Illumina nova6000 platform. RNA-seq reads were trimmed and qualified with fastp v0.23.4 (Chen et al., 2018) and subsequently mapped to reference genome using HISAT2 v2.2.1 (Kim et al., 2019). We use StringTie v2.2.3 (Pertea et al., 2015) to assembly the sequence and calculate the TPM values and obtained expression matrix with ballgown v2.36.0. DESeq2 v1.44.0 was used for differential expressed gene analysis. log (TPM+1) value was used for co-expression analysis.

**REFERENCES**

**Cantalapiedra, C.P., Ana, H.-P., Ivica, L., Peer, B., and Jaime, H.C.** (2021). eggNOG-mapper v2: Functional annotation, orthology assignments, and domain prediction at the metagenomic scale. Mol. Biol. Evol. **38:** 5825-5829.

**Capella-Gutiérrez, S., Silla-Martínez, J.M., and Gabaldón, T.** (2009). TrimAl: a tool for automated alignment trimming in large-scale phylogenetic analyses. Bioinformatics **25**.

**Chen, S., Zhou, Y., Chen, Y., and Gu, J.** (2018). Fastp: an ultra-fast all-in-one FASTQ preprocessor. Bioinformatics **34:** i884-i890.

**Cheng, C.Y., Krishnakumar, V., Chan, A.P., Thibaud-Nissen, F., and Schobel, S.** (2017). Araport11: a complete reannotation of the *Arabidopsis thaliana* reference genome. Plant J. **89:** 789-804.

**Cheng, H., Concepcion, G.T., Feng, X., Zhang, H., and Li, H.** (2021). Haplotype-resolved de novo assembly using phased assembly graphs with hifiasm. Nat. Methods **18:** 170-175.

**Consortium, T.G.** (2012). The tomato genome sequence provides insights into fleshy fruit evolution. Nature **485:** 635-641.

**Darriba, D., Posada, D., Kozlov, A.M., Stamatakis, A., Morel, B., and Flouri, T.** (2020). ModelTest-NG: a new and scalable tool for the selection of DNA and protein evolutionary models. Mol. Biol. Evol. **37:** 291-294.

**Gabriel, L., Brna, T., Hoff, K.J., Ebel, M., Lomsadze, A., Borodovsky, M., and Stanke, M.** (2024). BRAKER3: Fully automated genome annotation using RNA-seq and protein evidence with GeneMark-ETP, AUGUSTUS, and TSEBRA. Genome Res. **34:** 769-777.

**Gonda, I., Faigenboim, A., Adler, C., Milavski, R., Karp, M.-J., Shachter, A., Ronen, G., Baruch, K., Chaimovitsh, D., and Dudai, N.** (2020). The genome sequence of tetraploid sweet basil, *Ocimum basilicum* L., provides tools for advanced genome editing and molecular breeding. DNA Res. **27:** dsaa027.

**Haas, B.J., Salzberg, S.L., Zhu, W., Pertea, M., Allen, J.E., Orvis, J., White, O., Buell, C.R., and Wortman, J.R.** (2008). Automated eukaryotic gene structure annotation using EVidenceModeler and the Program to Assemble Spliced Alignments. Genome Biol. **9:** R7.

**Hamilton, J.P., Godden, G.T., Lanier, E., Bhat, W.W., and Buell, C.R.** (2020). Generation of a chromosome-scale genome assembly of the insect-repellent terpenoid-producing Lamiaceae species, *Callicarpa americana*. GigaScience **9:** giaa093.

**Huerta-Cepas, J., Szklarczyk, D., Heller, D., Hernández-Plaza, A., Forslund, S.K., Cook, H., Mende, D.R., Letunic, I., Rattei, T., and Jensen, L.J.** (2019). EggNOG 5.0: a hierarchical, functionally and phylogenetically annotated orthology resource based on 5090 organisms and 2502 viruses. Nucleic Acid Res. **47:** D309-D314.

**Jin, J., Tian, F., Yang, D., Meng, Y., Kong, L., Luo, J., and Gao, G.** (2017). PlantTFDB 4.0: toward a central hub for transcription factors and regulatory interactions in plants. Nucleic Acid Res. **45:** D1040-D1045.

**Jones, P., Binns, D., Chang, H.-Y., Fraser, M., Li, W., Mc Anulla, C., Mc William, H., Maslen, J., Mitchell, A., and Nuka, G.** (2014). InterProScan 5: genome-scale protein function classification. Bioinformatics **30:** 1236-1240.

**Katoh, K., Misawa, K., Kuma, K., and Miyata, T.** (2002). MAFFT: a novel method for rapid multiple sequence alignment based on fast Fourier transform. Nucleic Acid Res. **30:** 3059-3066.

**Kim, D., Paggi, J.M., Park, C., Bennett, C., and Salzberg, S.L.** (2019). Graph-based genome alignment and genotyping with HISAT2 and HISAT-genotype. Nat. Biotechnol. **37:** 907-915.

**Kozlov, A.M., Diego, D., Tomáš, F., Benoit, M., and Alexandros, S.** (2019). RAxML-NG: A fast, scalable, and user-friendly tool for maximum likelihood phylogenetic inference. Bioinformatics **35:** 4453-4455.

**Krzywinski, M., Schein, J., Birol, I., Connors, J., Gascoyne, R., Horsman, D., Jones, S.J., and Marra, M.A.** (2009). Circos: An information aesthetic for comparative genomics. Genome Res. **19:** 1639-1645.

**Li, H.** (2023). Protein-to-genome alignment with miniprot. Bioinformatics **39:** btad014.

**Li, J., Li, H., Wang, Y., Zhang, W., Wang, D., Dong, Y., Ling, Z., Bai, H., Jin, X., Hu, X., and Shi, L.** (2023). Decoupling subgenomes within hybrid lavandin provide new insights into speciation and monoterpenoid diversification of *Lavandula*. Plant Biotechnol. J. **21:** 2084-2099.

**Manni, M., Berkeley, M.R., Seppey, M., and Zdobnov, E.M.** (2021). BUSCO: Assessing genomic data quality and beyond. Curr. Protoc. **1:** e323.

**Mendes, F.K., Vanderpool, D., Fulton, B., and Hahn, M.W.** (2020). CAFE 5 models variation in evolutionary rates among gene families. Bioinformatics **36:** 5516-5518.

**Nolan, B., Hamilton, J.P., Pan, L., Wood, J.C., Natalia, D., and Robin, B.C.** (2020). Genome sequencing of four culinary herbs reveals terpenoid genes underlying chemodiversity in the Nepetoideae. DNA Res. **27:** dsaa016.

**Ou, S., Su, W., Liao, Y., Chougule, K., Agda, J.R.A., Hellinga, A.J., Lugo, C.S.B., Elliott, T.A., Ware, D., Peterson, T., Jiang, N., Hirsch, C.N., and Hufford, M.B.** (2019). Benchmarking transposable element annotation methods for creation of a streamlined, comprehensive pipeline. Genome Biol. **20:** 275.

**Pei, T., Zhu, S., Liao, W., Fang, Y., Liu, J., Kong, Y., Yan, M., Cui, M., and Zhao, Q.** (2023). Gap-free genome assembly and *CYP450* gene family analysis reveal the biosynthesis of anthocyanins in *Scutellaria baicalensis*. Hortic. Res. **10:** uhad235.

**Pertea, M., Pertea, G.M., Antonescu, C.M., Chang, T.C., Mendell, J.T., and Salzberg, S.L.** (2015). StringTie enables improved reconstruction of a transcriptome from RNA-seq reads. Nat. Biotechnol. **33:** 290-295.

**Salojärvi, J., Rambani, A., Yu, Z., Guyot, R., Strickler, S., Lepelley, M., Wang, C., Rajaraman, S., Rastas, P., Zheng, C., Muñoz, D.S., Meidanis, J., Paschoal, A.R., and Bawin, Y.** (2024). The genome and population genomics of allopolyploid *Coffea arabica* reveal the diversification history of modern coffee cultivars. Nat. Genet. **56:** 721-731.

**Smit, S.J., Ayten, S., Radzikowska, B.A., Hamilton, J.P., Langer, S., Unsworth, W.P., Larson, T.R., Buell, C.R., and Lichman, B.R.** (2024). The genomic and enzymatic basis for iridoid biosynthesis in cat thyme (*Teucrium marum*). Plant J. **118:** 1589-1602.

**Stanke, M., Keller, O., Gunduz, I., Hayes, A., Waack, S., and Morgenstern, B.** (2006). AUGUSTUS: ab initio prediction of alternative transcripts. Nucleic Acid Res **34:** W435-W439.

**Stiehler, F., Steinborn, M., Scholz, S., Dey, D., Andreas P M, W., and Alisandra K, D.** (2020). Helixer: cross-species gene annotation of large eukaryotic genomes using deep learning. Bioinformatics **36:** 5291-5298.

**Sun, M., Zhang, Y., Zhu, L., Liu, N., Bai, H., Sun, G., Zhang, J., and Shi, L.** (2022). Chromosome-level assembly and analysis of the Thymus genome provide insights into glandular secretory trichome formation and monoterpenoid biosynthesis in thyme. Plant Commun. **3:** 100413.

**Sun, P., Jiao, B., Yang, Y., Shan, L., Li, T., Li, X., Xi, Z., Wang, X., and Liu, J.** (2021). WGDI: A user-friendly toolkit for evolutionary analyses of whole-genome duplications and ancestral karyotypes. Mol. Plant **15:** 1841-1851.

**Suyama, M., Torrents, D., and Bork, P.** (2006). PAL2NAL: robust conversion of protein sequence alignments into the corresponding codon alignments. Nucleic Acid Res. **34:** W609-W612.

**Tamura, K., Stecher, G., and Kumar, S.** (2021). MEGA11: Molecular Evolutionary Genetics Analysis Version 11. Mol. Biol. Evol. **38:** 3022-3027.

**Tang, H., Krishnakumar, V., Zeng, X., Xu, Z., Taranto, A., Lomas, J.S., Zhang, Y., Huang, Y., Wang, Y., and Yim, W.C.** (2024). JCVI: A versatile toolkit for comparative genomics analysis. iMeta **3:** e211.

**Wang, Y., Li, J., and Andrew H, P.** (2013). MCScanX-transposed: detecting transposed gene duplications based on multiple colinearity scans. Bioinformatics **29:** 1458-1460.

**Yang, Z.** (2007). PAML 4: Phylogenetic analysis by maximum likelihood. Mol. Biol. Evol. **24:** 1586-1591.

**Zeng, X., Yi, Z., Zhang, X., Du, Y., Li, Y., Zhou, Z., Chen, S., Zhao, H., Yang, S., Wang, Y., and Chen, G.** (2024). Chromosome-level scaffolding of haplotype-resolved assemblies using Hi-C data without reference genomes. Nat. Plants **10:** 1184-1200.

**Zhang, Y., Shen, Q., Leng, L., Zhang, D., Chen, S., Shi, Y., Ning, Z., and Chen, S.** (2021). Incipient diploidization of the medicinal plant Perilla within 10,000 years. Nat. Commun. **12:** 5508.
