# Supplementary material for: Integrative chromosome-level genomics and metabolomics uncover regulatory networks linking monoterpenoid biosynthesis and glandular trichome formation in Mosla chinensis
Source: Hortic Res. 2025 Oct 1;13(1):uhaf263. doi: 10.1093/hr/uhaf263 (PMC12861481; doi:10.1093/hr/uhaf263)
Supplement: Web_Material_uhaf263 [file web_material_uhaf263.zip › Supplemental Table.docx]

**Table S1 Comparison of agronomic traits between Mc and McJ**

|  | ***Mosla chinensi*s Maxim. (Mc)** | | | | | ***Mosla chinensis* 'Jiangxiangru' (McJ)** | | | | |
| --- | --- | --- | --- | --- | --- | --- | --- | --- | --- | --- |
|  | **Plant height (cm)** | **Plant width (cm)** | **Stem diameter (mm)** | **Leaf length (mm)** | **Leaf width (cm)** | **Plant height (cm)** | **Plant width (cm)** | **Stem diameter (mm)** | **Leaf length (mm)** | **Leaf width (cm)** |
| 1 | 14 | 8.5 | 1.12 | 16.58 | 2.11 | 67.5 | 59 | 6.17 | 27.12 | 6.25 |
| 2 | 15.5 | 10.6 | 1.23 | 14.78 | 2.46 | 72.4 | 44.3 | 5.37 | 24.61 | 5.89 |
| 3 | 15.3 | 13.2 | 1.2 | 16.58 | 2.58 | 89.9 | 55.6 | 6.11 | 34.43 | 6.97 |
| 4 | 14.5 | 20.5 | 1.68 | 18.20 | 3.36 | 84.2 | 68.5 | 7.7 | 33.51 | 6.09 |
| 5 | 20.6 | 11.5 | 1.14 | 20.14 | 2.74 | 82.5 | 54.4 | 5.01 | 27.35 | 6.05 |
| 6 | 25.4 | 13.3 | 1.16 | 18.17 | 3.06 | 96.3 | 77.4 | 5.63 | 32.47 | 5.76 |
| 7 | 17.2 | 8.8 | 0.77 | 16.08 | 2.62 | 96.3 | 63.7 | 4.96 | 27.85 | 6.73 |
| 8 | 21.3 | 18.5 | 1.44 | 15.64 | 2.77 | 101.0 | 54.5 | 6.7 | 27.00 | 5.72 |
| 9 | 15.1 | 13.2 | 1.82 | 16.19 | 3.21 | 85.5 | 83.6 | 6.27 | 29.44 | 5.00 |
| 10 | 20.9 | 12.5 | 1.37 | 14.69 | 2.71 | 100.3 | 73.2 | 5.11 | 29.65 | 6.14 |
| 11 | 15.3 | 9.9 | 1.39 | 20.08 | 2.85 | 88.6 | 63.7 | 5.8 | 37.08 | 6.38 |
| 12 | 25.1 | 12.8 | 1.31 | 14.30 | 3.25 | 84.4 | 79.6 | 7.24 | 29.99 | 5.94 |
| 13 | 14.8 | 8.6 | 0.94 | 14.25 | 2.57 | 77.4 | 88.7 | 9.28 | 31.33 | 7.81 |
| 14 | 25.5 | 25.5 | 1.54 | 22.87 | 3.11 | 101.4 | 83.2 | 4.37 | 29.81 | 6.32 |
| 15 | 29.4 | 10.9 | 1.07 | 16.94 | 1.86 | 87.2 | 55 | 6.81 | 30.20 | 6.47 |
| 16 | 15.4 | 5.2 | 1.27 | 11.59 | 2.45 | 79.6 | 51.2 | 4.02 | 34.44 | 5.63 |
| 17 | 8.9 | 4.8 | 1.05 | 17.85 | 2.88 | 79.1 | 55.7 | 4.57 | 29.85 | 6.29 |
| 18 | 12.5 | 11 | 0.94 | 18.19 | 3.23 | 103.1 | 60.5 | 7.69 | 33.92 | 6.24 |
| 19 | 13.8 | 7.2 | 0.83 | 14.97 | 2.83 | 94.1 | 78.6 | 5.53 | 32.24 | 5.63 |
| 20 | 13.4 | 6.8 | 0.96 | 15.70 | 2.79 | 74.3 | 54.4 | 5.77 | 32.07 | 6.07 |
| 21 | 13.9 | 7.5 | 1.02 | 16.89 | 2.87 | 101.2 | 74.4 | 4.54 | 30.11 | 5.96 |
| 22 | 12.1 | 7.3 | 1.07 | 15.55 | 2.67 | 94.9 | 57.4 | 5.51 | 34.79 | 5.50 |
| 23 | 13.4 | 5.7 | 0.79 | 20.20 | 3.20 | 99.6 | 73.3 | 6.1 | 29.95 | 6.05 |
| 24 | 16.8 | 9.8 | 1.26 | 16.86 | 3.17 | 97.4 | 43 | 5.09 | 26.87 | 5.87 |

**Table S2 Trichome numbers in Mc and McJ**

|  | ***Mosla chinensis* Maxim. (Mc)** | ***Mosla chinensis* 'Jiangxiangru' (McJ)** |
| --- | --- | --- |
| 1 | 37 | 32 |
| 2 | 39 | 36 |
| 3 | 42 | 28 |
| 4 | 40 | 31 |
| 5 | 44 | 32 |
| 6 | 42 | 35 |
| 7 | 38 | 40 |
| 8 | 42 | 30 |
| 9 | 45 | 40 |
| 10 | 43 | 27 |
| 11 | 43 | 29 |
| 12 | 39 | 28 |
| 13 | 39 | 33 |
| 14 | 39 | 36 |
| 15 | 39 | 37 |
| 16 | 49 | 44 |
| 17 | 56 | 51 |
| 18 | 63 | 37 |
| 19 | 49 | 36 |
| 20 | 48 | 39 |
| 21 | 45 | 38 |
| 22 | 49 | 41 |
| 23 | 49 | 44 |
| 24 | 44 | 32 |

**Table S3 Carvacrol and thymol content in Mc and McJ**

|  | ***Mosla chinensis* Maxim. (Mc)** | | ***Mosla chinensis* 'Jiangxiangru' (McJ)** | |
| --- | --- | --- | --- | --- |
|  | **Carvacrol (mg/g)** | **Thymol (mg/g)** | **Carvacrol (mg/g)** | **Thymol (mg/g)** |
| 1 | 0.862710976 | 2.64101323 | 0.689879161 | 2.194015113 |
| 2 | 0.558427117 | 1.784349203 | 0.217543901 | 2.281528116 |
| 3 | 0.804684947 | 2.520643346 | 0.575831605 | 1.105493339 |
| 4 | 0.522492066 | 1.657474762 | 0.512454863 | 1.620286764 |
| 5 | 0.60567682 | 1.958940663 | 0.691198011 | 2.165515206 |
| 6 | 0.6687264 | 2.12897781 | 0.720632331 | 2.255854552 |

**Table S4 Statistics of sequencing data**

| **Species** | **platform** | **num_seqs** | **sum_len** | **avg_len** | **max_len** | **N50** | **N50_num** | **AvgQual** |
| --- | --- | --- | --- | --- | --- | --- | --- | --- |
| *M. chinensis* | PacBio HiFi | 1851929 | 32410634238 | 17501 | 53265 | 17999 | 16602 | 22.87 |
| M. chinensis J. | PacBio HiFi | 1993294 | 39009843105 | 19570.5 | 50393 | 19875 | 21144 | 26.85 |
| *M. soochowensis* | PacBio HiFi | 2069197 | 41977937591 | 20287.1 | 50087 | 20747 | 21713 | 26.96 |
| *M. chinensis* | HiC R1 | 248069733 | 37120964787 | 149.6 | 150 | - | - | - |
|  | HiC R2 | 248069733 | 37120964787 | 149.6 | 150 | - | - | - |
| M. chinensis J. | HiC R1 | 291578351 | 43636116984 | 149.7 | 150 | - | - | - |
|  | HiC R2 | 291578351 | 43636807753 | 149.7 | 150 | - | - | - |

**Table S5 Classification of repeat sequences of genome**

| **Species** | **Mc** | | | **McJ** | | | **Ms** | | |
| --- | --- | --- | --- | --- | --- | --- | --- | --- | --- |
|  | Number | Length (bp) | % | Number | Length (bp) | % | Number | Length (bp) | % |
| **Retroelements** | 245,310 | 139,652,134 | 32.77 | 217,202 | 125,029,672 | 30.58 | 257,226 | 139,680,213 | 33.84 |
| SINEs | 17,842 | 2,189,007 | 0.51 | 16,146 | 2,039,692 | 0.5 | 11,707 | 1,389,406 | 0.34 |
| Penelope | 884 | 104,203 | 0.02 | 953 | 140,729 | 0.03 | 0 | 0 | 0 |
| LINEs | 4,386 | 2,038,251 | 0.48 | 6,195 | 1,997,838 | 0.49 | 3,801 | 1,888,820 | 0.46 |
| CRE/SLACS | 0 | 0 | 0 | 0 | 0 | 0 | 0 | 0 | 0 |
| L2/CR1/Rex | 0 | 0 | 0 | 0 | 0 | 0 | 0 | 0 | 0 |
| R1/LOA/Jockey | 0 | 0 | 0 | 0 | 0 | 0 | 0 | 0 | 0 |
| R2/R4/NeSL | 0 | 0 | 0 | 0 | 0 | 0 | 0 | 0 | 0 |
| RTE/Bov-B | 0 | 0 | 0 | 0 | 0 | 0 | 0 | 0 | 0 |
| L1/CIN4 | 3,502 | 1,934,048 | 0.45 | 3,644 | 1,768,424 | 0.43 | 3,801 | 1,888,820 | 0.46 |
| **LTR elements** | 223,082 | 135,424,876 | 31.78 | 194,861 | 120,992,142 | 29.6 | 241,718 | 136,401,987 | 33.04 |
| BEL/Pao | 0 | 0 | 0 | 0 | 0 | 0 | 7 | 4646 | 0 |
| Ty1/Copia | 25,196 | 22,962,416 | 5.39 | 30,802 | 23,810,169 | 5.82 | 23,911 | 18,570,621 | 4.5 |
| Gypsy/DIRS1 | 70,710 | 36,939,107 | 8.67 | 59,331 | 38,440,573 | 9.4 | 74,839 | 37,624,365 | 9.11 |
| Retroviral | 0 | 0 | 0 | 0 | 0 | 0 | 0 | 0 | 0 |
| **DNA transposons** | 356,391 | 90,682,369 | 21.28 | 354,656 | 92,600,008 | 22.65 | 295,651 | 76,179,947 | 18.45 |
| hobo-Activator | 199 | 50,768 | 0.01 | 317 | 102,362 | 0.03 | 647 | 205,009 | 0.05 |
| Tc1-IS630-Pogo | 0 | 0 | 0 | 373 | 48,710 | 0.01 | 0 | 0 | 0 |
| En-Spm | 0 | 0 | 0 | 0 | 0 | 0 | 0 | 0 | 0 |
| MuDR-IS905 | 0 | 0 | 0 | 0 | 0 | 0 | 0 | 0 | 0 |
| PiggyBac | 0 | 0 | 0 | 0 | 0 | 0 | 0 | 0 | 0 |
| Tourist/Harbinger | 349 | 173,782 | 0.04 | 837 | 323,952 | 0.08 | 604 | 251,039 | 0.06 |
| Other | 0 | 0 | 0 | 0 | 0 | 0 | 0 | 0 | 0 |
| **Rolling-circles** | 1,548 | 392,072 | 0.09 | 465 | 183,012 | 0.04 | 507 | 399,641 | 0.1 |
| **Unclassified** | 59,698 | 13,284,744 | 3.12 | 58,297 | 12,651,695 | 3.09 | 64,424 | 14,621,064 | 3.54 |
| **Total** |  | 243,619,247 | 57.17 |  | 230,281,375 | 56.33 |  | 230,481,224 | 55.83 |

**Table S6 Statistics of orthologous genes of the species selected for phylogenomic analysis**

| **Species** | **Genes** | | | **Families** | |
| --- | --- | --- | --- | --- | --- |
|  | **Total** | **Within-families** | **Unassigned** | **Total** | **Unique** |
| *Arabidopsis thaliana* | 27445 | 24947 | 2498 | 13988 | 916 |
| *Solanum lycopersicum* | 34075 | 29434 | 4641 | 15984 | 823 |
| *Coffea canephora* | 25574 | 23435 | 2139 | 15365 | 490 |
| *Callicarpa americana* | 32164 | 29994 | 2170 | 17070 | 476 |
| *Scutellaria baicalensis* | 28080 | 26465 | 1615 | 15943 | 495 |
| *Teucrium marum* | 36053 | 32234 | 3819 | 16565 | 1154 |
| Lavandin | 58702 | 52630 | 6072 | 18100 | 1399 |
| *Ocimum basilicum* | 65635 | 61075 | 4560 | 18651 | 2211 |
| *Origanum majorana* | 27795 | 26863 | 932 | 16536 | 204 |
| *Origanum vulgare* | 26753 | 25976 | 777 | 16382 | 154 |
| *Thymus quinquecostatus* | 29676 | 29174 | 502 | 16457 | 224 |

**Table S7 Amino acid sequence of TPSs in Mc, McJ and 11 Labiatae plants**

| **Name** | **Sequence** |
| --- | --- |
| McTPS1 | MYSISSNVNVVPILSNSHTRRPSKPSRVSSGAPPARLRCTCSSQLDVKPVDEIAPVARRCGNYQPSIWDINFIESLNTTQYREESNLKRKEELIEQVKLMLDQKMDAVQQLALIEDLRNVGLTYFFQDQIKKILTSIYNENKCFQGINIDQAEEKGLYFTALGFRLLRYHGFQVSQEVFDCFKNEEGSDFKASLSDDTQALLQLYEASFLLREGEDTLELARQFSTKFIRKKLDDGSIDDSNLVSWIRHSLALPLHWRIQWLEARWFLDAYAARHDRNPVIFELAKLDFNIIQEIHMEEIKDVSRWWSGSSLAEKLPFVRDRIVECYFWAVGLFEPHEYRYQRIMATKIITFVTIIDDVYDVYGTLDELQLFTDTVRRWDTESISQLPYYMQVCYLALYNFATDLAYDILKDNGIIAIPYLQQSWLDLVSGFFVEAKWFYSGYTPTLEEYLKNGRVSISSPSIISQVYFTLPSTISPKKEEIDTLFKYHDIIYLSGMILRLADDLGTTPFELKRGDVPKAIQCYMKDTNCTEEEAQEHVRHLIRESWKEMNTVSASADCPFSDDLVDAAAHLGRVAQFMYLDGDGHGVQHSEIHHQMAGLMLEPYI |
| McTPS2 | MCSIGMHVVPILASNPCLITTKASKPWRVSASVTRLRSSCSSQLDVDQIATARRSGNYQPSLWDFNYLQSLNTTHYKEERHLKREAALIEQVKMLLEEEMGVVQQLELVDDLKNLGLTYFFEDQIKQILTFIYNDHKCFHGNNIIEAEERGLYFTALGFRLLRQYGFQVSQEVFDSFKNEDGSDFKASLDDDTKGLLQLYEASFLLREGEDTLELARKFATNFLEKKVDDHQLIDDTNLLSRISHSLELPLHWRIQWLEARWFLNAYATRHDMNPIIFELAKLDFNIIQATHQEEIKHVSRWWNSSGLAEKLPFVRDRIVECYFWAVGLFEPHEYGYQRIIAAKIITFVTIIDDIYDVYGTLDELQLFTNTIRRWDTESMSELPYYMQLCYLALYNFASEMAYNILNDKGVITIPYLQRSWVDLVEGFFEEAKWYYSGYTPSLEEYMNNGKVSISSPTIISQIYFTLAATSPHKKDDIDILYKYHHILYLSGIILRLADDLGTAPFELKRGDVPKAIQCYMKDTNGTEKEAQEHVRFLIREAWKEMNTAAAAADCPFSGDLVAAAANLGRTAQFIYVDGDGHGVQHSQIHQQMAGLMFQPYTSEIGD |
| McTPS3 | MCSMRIHVAILNKAIKDVKASKPSWRVSSSSRASCSLQLMNVKPAHQIRRSGNYRPSLWDFNYLQSLDATHYKEERYLKREAELIEQVKMLLDHEEMEATQQLELVDDLKNLGLSYFFEDQIKQILMLIYNEHKCFHGNNIEAEERGLYFTALGFRLLRQHGFQVSQELFDSFKDEEGSDFKASFGDDIEGLLQLYEASFLLREGEDTLELARKFATNFLEKKVDDHQLIDDTNLLSCIRHSLEIPLHWRIQRLEARWFLDAYATRHDMNPIILELANLDFNIIQATQQEELKELSRWWKSTGLAEKLPFVRDRLVESYFWAVALFEPHHYGYHRKIAAKIITLITSLDDVYDIYGTLDELQLFTDVIQRWDTESINRLPYYMQLFYMVLYNFVSELAYDGLKEKGFITLPYLQRSWADLVEAYLKEAKWYHNGYTPSMEEYLNNAYISIGATPVISQVFFTLATSIDKPVIESLYEYHPILRLSGMLVRLPDDLGTSPFEMKRGDVPKAIQLYMKERNATEKEAQEHVRFLIREAWKEMNTATAATDCPFTDDLVAAAANLGRAAQFMYLDGDGNHSQLHQQIACLLFEPYA |
| McTPS4 | MRSVLLQVAIVKPAYYLHYSSNNIVSPIAAAATTRRLRPHYFSIHFSDQTQTGRRSGGYQPALWDFDSIQSLYNEYKEEVHRTRAQRLIDQVKMLLLEEVDHVRQLELIEDLHRLGVSCHFEKEIIQILNLIYLNYETDQRDLYSTSLGFRLLRQHGFNVSQEVFDCFKNDEGTGFKRSLSRDIKGLLQLYEASFLLTQGENTLELAREFATKFLQEKLDQDQIDDDDDDDDDYLVSLIRHALEFPSHWRVQMPNAISFIDAYKRRPDMNPTVLELAKLDINIVQAQFQDELKETSRWWESTCLVQQLPFVRDRIVECYLWTTGVIQRREHGYERIMLTKINALVTTIDDVFDIYGTVEELHLFTNAIQSWDLESMKQLPSYMQLCYLALYNFINEMAYATLVEKGFNSIPYLRKTWVDLIESYLIEAKWYYDGHKPTLEEYMNNAWISIGGVPILSHIFFRLTDPIEKEGTVESMHKYHDVVRASCTILRLADDMGTSLDEVERGDVPKSIQCYKNEKNASEEEAREHVRSLMAETWKTINHEMIDSPFSKYFVEALANLARMAQFIYQDGSDGFGMQHSKVNKLLRGLLFDRYA |
| McTPS5 | MSSIILMHTAIPNNKPSKYVDNIISKQNPQLRRSSPTCMRRRRPSSLQLHASYQLESPPRRTGNYKPTLWDFDRIQSLNSVYKEEKYTTRASELMVQVKKLLEAESDWFGQLELIDQLQKLGLSYHFNEEINQILNSIYFEQKYYSKAEERDLYSTSLAFRLLRQHGLKVSQDVFDGFKNDKGDFEASLGDDIEGVQQMYEASFLLVEGEMTMEAARVFSTEILQRKLDDGGVDDDEQLSALVRRSLELPLHWCVQRPNARWFIEAYSTRSDANTALLELAKLDFNIVQAEHQKELKQISRWWKETRLAEKLPFARDRLVENYIWNIGLLFEPQYGHARIMTTKLFVLITVIDDVFDVYGTLEETQLFNDTILRWDVEAIEKLPEYMQICYMALDNFINETAYDILKEHGILTIQDLRKSWSDLCSAYAKEAEWYHTEYTPTLDEYMSVSWISISAHTILLWVFFFITNPLEKDVIQKWRNYHEIEIIRCSAFVLRLADDLGTSPFELKRGDVPKAVECYMNERGASREEAKQHVWNMLWDTWKKMNEENFGDSPFSKDFMRSAADLGRQAQYMYQHGDGHGIRNREMEERILGLIFNPIV |
| McJTPS1 | MYSISSNVNVVPILSNSHTRRPSKPSRVSSGAPPARLRCTCSSQLDVKPVDEIAPVARRCGNYQPSIWDINFIESLNTTQYREESNLKRKEELIEQVKLMLDQKMDAVQQLALIEDLRNVGLTYFFQDQIKKILTSIYNENKCFQGINIDQAEEKGLYFTALGFRLLRYHGFQVSQEVFDCFKNEEGSDFKASLSDDTQALLQLYEASFLLREGEDTLELARQFSTKFIRKKLDDGSIDDSNLVSWIRHSLALPLHWRIQWLEARWFLDAYAARHDRNPVIFELAKLDFNIIQEIHMEEIKDVSRWWSGSSLAEKLPFVRDRIVECYFWAVGLFEPHEYRYQRIMATKIITFVTIIDDVYDVYGTLDELQLFTDTVRRWDTESISQLPYYMQVCYLALYNFATDLAYDILKDNGIIAIPYLQQSWLDLVSGFFVEAKWFYSGYTPTLEEYLKNGRVSISSPSIISQVYFTLPSTISPKKEEIDTLFKYHDIIYLSGMILRLADDLGTTPFELKRGDVPKAIQCYMKDTNCTEEEAQEHVRHLIRESWKEMNTVSASADCPFSDDLVDAAAHLGRVAQFMYLDGDGHGVQHSEIHHQMAGLMLEPYI |
| McJTPS2 | MCSIGMHVVPILASNPCLITTKASKPWRVSASVTRLRSSCSSQLDVDQIATARRSGNYQPSLWDFNYLQSLNTTHYKEERHLKREAALIEQVKMLLEEEMGVVQQLELVDDLKNLGLTYFFEDQIKQILTFIYNDHKCFHGNNIIEAEERGLYFTALGFRLLRQYGFQVSQEVFDSFKNEDGSDFKASLDDDTKGLLQLYEASFLLREGEDTLELARKFATNFLEKKVDDHQLIDDSNLLSRISHSLELPLHWRIQWLEARWFLNAYATRHDMNPIIFELAKLDFNIIQATHQEEIKHVSRWWNSSGLAEKLPFVRDRIVECYFWAVGLFEPHEYGYQRIIAAKIITFVTIIDDIYDVYGTLDELQLFTNTIRRWDTESMSELPYYMQLCYLALYNFASEMAYNILNDKGVITIPYLQRSWVDLVEGFFEEAKWYYSGYTPSLEEYMNNGKVSISSPTIISQIYFTLAATSPHKKDDIDILYKYHHILYLSGIILRLADDLGTAPFELKRGDVPKAIQCYMKDTNGTEKEAQEHVRFLIREAWKEMNTAAAAADCPFSGDLVAAAANLGRTAQFIYVDGDGHGVQHSQIHQQMAGLMFQPYTSEIGD |
| McJTPS3 | MCSMRIHVAILNKAIKDVKASKPSWRVSSSSRASCSLQLMNVKPAHQIRRSGNYRPSLWDFNYLQSLDATHYKEERYLKREAELIEQVKMLLDHEEMEATQQLELVDDLKNLGLSYFFEDQIKQILMLIYNEHKCFHGNNIEAEERGLYFTALGFRLLRQHGFQVSQELFDSFKDEEGSDFKASFGDDIEGLLQLYEASFLLREGEDTLELARKFATNFLEKKVDDHQLIDDTNLLSCIRHSLEIPLHWRIQRLEARWFLDAYATRHDMNPIILELANLDFNIIQATQQEELKELSRWWKSTGLAEKLPFVRDRLVESYFWAVALFEPHHYGYHRKIAAKIITLITSLDDVYDIYGTLDELQLFTDVIQRWDTESINRLPYYMQLFYMVLYNFVSELAYDGLKEKGFITLPYLQRSWADLVEAYLKEAKWYHNGYTPSMEEYLNNAYISIGATPVISQVFFTLATSIDKPVIESLYEYHPILRLSGMLVRLPDDLGTSPFEMKRGDVPKAIQLYMKERNATEKEAQEHVRFLIREAWKEMNTATAATDCPFTDDLVAAAANLGRAAQFMYLDGDGNHSQLHQQIACLLFEPYA |
| McJTPS4 | MRSVLLQVAIVKPAYYLHYSSNNIVSPIAAAATTRRLRPHYFSIHFSDQTQTGRRSGGYQPALWDFDSIQSLYNEYKEEVHRTRAQRLIDQVKMLLLEEVDHVRQLELIEDLHRLGVSCHFEKEIIQILNLIYLNYETDQRDLYSTSLGFRLLRQHGFNVSQELFDCFKNDEGTGFKRSLARDIKGLLQLYEASFLLTQGENTLELAREFATKFLQEKLDQDQIDDDDDDYLVSLIRHALEFPSHWRVQMPNAISFIDAYKRRPDMNPTVLELAKLDINIVQAQFQDELKETSRWWESTCLVQQLPFVRDRIVECYLWTTGVIQRREHGYERIMLTKINALVTTIDDVFDIYGTVEELHLFTNAIQSWDLESMKQLPSYMQLCYLALYNFINETAYATLVEKGFNSIPYLRKTWVDLIESYLIEAKWYYDGHKPTLEEYMNNAWISIGGVPILSHIFFRLTDPIEKEGTVESMHKYHDVVRASCTILRLADDMGTSLDEVERGDVPKSIQCYMNEKNASEEEAREHVRSLMAETWKTINHEMIDSPFSKYFVEALANLARMAQFIYQDGSDGFGMQHSKVNKLLRGLLFDRYA |
| McJTPS5 | MSSIILMHTAIPNNKPSKYVDNIISKQNPQLRRSSPTCMRRRRPSSLQLHASYQLESPPRRTGNYKPTLWDFDRIQSLNSVYKEEKYTTRASELMVQVKKLLEAESDWFGQLELIDQLQKLGLSYHFNEEINQILNSIYFEQKYYSKAEERDLYSTSLAFRLLRQHGLKVSQDVFDGFKNDKGDFEASLGDDIEGVQQMYEASFLLVEGEMTMEAARVFSTEILQRKLDDGGVDDDEQLSALVRRSLELPLHWCVQRPNARWFIEAYSTRSDANTALLELAKLDFNIVQAEHQKELKQISRWWKETRLAEKLPFARDRLVENYIWNIGLLFEPQYGHARIMTTKLFVLITVIDDVFDVYGTLEETQLFNDTILRWDVEAIEKLPEYMQICYMALDNFINETAYDILKEHGILTIQDLRKSWSDLCSAYAKEAEWYHTEYTPTLDEYMSVSWISISAHTILLWVFFFITNPLEKDVIQKWRNYHEIEIIRCSAFVLRLADDLGTSPFELKRGDVPKAVECYMNERGASREEAKQHVWNMLWDTWKKMNEENFGDSPFSKDFMRSAADLGRQAQYMYQHGDGHGIRNREMEERILGLIFNPIV |
| SfCinS | MSSLIMQVVIPKPAKFFHNNLFSLSSKRHRFSTTTTTRGGRWARCSLQTGNEIQTERRTGGYQPTLWDFSTIQSFDSEYKEEKHLMRAAGMIDQVKMMLQEEVDSIRRLELIDDLRRLGISCHFEREIVEILNSKYYTNNEIDERDLYSTALRFRLLRQYDFSVSQEVFDCFKNAKGTDFKPSLVDDTRGLLQLYEASFLSAQGEETLRLARDFATKFLQKRVLVDKDINLLSSIERALELPTHWRVQMPNARSFIDAYKRRPDMNPTVLELAKLDFNMVQAQFQQELKEASRWWNSTGLVHELPFVRDRIVECYYWTTGVVERRQHGYERIMLTKINALVTTIDDVFDIYGTLEELQLFTTAIQRWDIESMKQLPPYMQICYLALFNFVNEMAYDTLRDKGFDSTPYLRKVWVGLIESYLIEAKWYYKGHKPSLEEYMKNSWISIGGIPILSHLFFRLTDSIEEEAAESMHKYHDIVRASCTILRLADDMGTSLDEVERGDVPKSVQCYMNEKNASEEEAREHVRSLIDQTWKMMNKEMMTSSFSKYFVEVSANLARMAQWIYQHESDGFGMQHSLVNKMLRDLLFHRYE |
| PfLIS | MSSMRTYVAIMKKPSVEHVDNVDKKASKPSWRVSLSAGLRSSCSLQLEVKPADQILTARRSGNYQPSLWDFNYLQSLNTTHYKEVRHLKREAELIEQVKMLLEEEMEAVQQLELVDDLKNLGLSYFFEDQIKQILTFIYNEHKCFHSNSIIEAEEIRDLYFTALGFRLLRQHGFQISQEVFDCFKNEEGSDFKARLGDDTKGLLQLYEASFLLREGEDTLELARQYATKFLQKKVDHELIDDNNLLSWILHSLEIPLHWRIQRLEARWFLDAYASRRDMNQIILELAKLDFNIIQATQQEELKDLSRWWKSSCLAEKLPFVRDRLVESYFWAIALFEPHQYGYHRKIAAKIITLITSLDDVYDIYGTLDELQLFTDAIQRWDTESISRLPYYMQLFYMVLYNFVPRLAYDGLKEKGFITIPYLQRSWADLVEAYLKEAKWYYNGYTPSMEEYLNNAYISIGATPVISQVFFTLATSIDKPVIDSLYEYHRILRLSGILVRLPDDLGTSPFEMKRGDVPKAIQLYMKERNATEIEAQEHVRFLIREAWKEMNTATAAVDCPFTDDLVTAAANLGRAAQFMYLDGDGNHSQLHQRIACLLFEPYA |
| SoCinS | MSSLIMQVVIPKPAKIFHNNLFSVISKRHRFSTTITTRGGRWAHCSLQMGNEIQTGRRTGGYQPTLWDFSTIQLFDSEYKEEKHLMRAAGMIAQVNMLLQEEVDSIQRLELIDDLRRLGISCHFDREIVEILNSKYYTNNEIDESDLYSTALRFKLLRQYDFSVSQEVFDCFKNDKGTDFKPSLVDDTRGLLQLYEASFLSAQGEETLHLARDFATKFLHKRVLVDKDINLLSSIERALELPTHWRVQMPNARSFIDAYKRRPDMNPTVLELAKLDFNMVQAQFQQELKEASRWWNSTGLVHELPFVRDRIVECYYWTTGVVERREHGYERIMLTKINALVTTIDDVFDIYGTLEELQLFTTAIQRWDIESMKQLPPYMQICYLALFNFVNEMAYDTLRDKGFNSTPYLRKAWVDLVESYLIEAKWYYMGHKPSLEEYMKNSWISIGGIPILSHLFFRLTDSIEEEDAESMHKYHDIVRASCTILRLADDMGTSLDEVERGDVPKSVQCYMNEKNASEEEAREHVRSLIDQTWKMMNKEMMTSSFSKYFVQVSANLARMAQWIYQHESDGFGMQHSLVNKMLRGLLFDRYE |
| MaLIS | MCTIISVNHHHVAILSKPKVKLFHTKNKRSASINLPWSLSPSSSAASRPISCSISSKLYTISSAQEETRRSGNYHPSVWDFDFIQSLDTDHYKEEKQLEREEELIMEVKKLLGAKMEATKQLELIDDLQNLGLSYFFRDEIKNILNSIYKIFQNNNSTKVGDLHFTSLGFRLLRQHGFNVSQGVFDCFKNEHGSDFEKTLIGEDTKGVLQLYEASFLLREGEDTLEVARKFSTEFLEEKLKAGIDGDNLSSSIGHSLEIPLHWRIQRLEERWFLDAYSRRKDMNPIIFELAKLDFNIIQATQQEELKDLSRWWNDSSLPQKLPFVRDRLVESYYWALGLFEAHKFGYERKTAAKIITLITALDDVYDIYGTLDELQLFTHVIRRWDTESATQLPYYLQLFYFVLYNFVSEVAYHILKEEGFISIPFLHRAWVDLVEGYLQEAKWYYTKYTPTMEEYLNYASITIGAPAVISQIYFMLAKSKEKPVIESFYEYDEIIRLSGMLVRLPDDLGTLPFEMKRGDVAKSIQIYMKEQNATREEAEEHVRFMIREAWKEMNTTMAANSDLRGDVVMAAANLGRDAQFMYLDGDGNHSQLQHRIANLLFKPYV |
| TvTPS1 | MRRSGNYQAPVWNNDFIQSFSTDKYKDEKFLKKKEELIAQVKVLLNTKMEAVKQLELIEDLRNLGLTYYFEDEFKKILTSIYNEHKGFKNEQVGDLYFTSLAFRLLRLHGFDVSEDVFNFFKNEDGSDFKASLGENTKDVLELYEASFLIRVGEVTLEQARVFSTKILEKKVEEGIKDEKLLAWIQHSLALPLHWRIQRLEARWFLDAYKARKDMNPIIYELGKIDFHIIQETQLQEVQEVSQWWTNTNLAEKLPFVRDRIVECYFWALGLFEPHEYGYQRKMAAIIITFVTIIDDVYDVYDTLDELQLFTDAIRKWDVESISTLPYYMQVCYLAVFTYASELAYDILKDQGFNSISYLQRSWLSLVEGFFQEAKWYYAGYTPTLAEYLENAKVSISSPTIISQVYFTLPNSTERTVVENVFGYHNILYLSGMILRLADDLGTTQFELKRGDVQKAIQCYMNDNNATEEEGTEHVKYLLREAWQEMNSAMADPDCPLSEDLVFAAANLGRTSQFIYLDGDGHGVQHSEIHNQMGGLIFEPYV |
| TvTPS2 | MATLSMQVSTLSKQVKNLNTFGMGSASKLPMVARRVSTTRLRPICSASLQVEEETRRSGNYQAPVWNNDFIQSFSTDKYKDEKFLKKKEELIAQVKVLLNTKMEAVKQLELIEDLRNLGLTYYFEDEFKKILTSIYNEHKGFKNEQVGDLYFTSLAFRLLRLHGFDVSEDVFNFFKNEDGSDFKASLGENTKDVLELYEASFLIRVGEVTLEQARVFSTKILEKKVEEGIKDEKLLAWIQHSLALPLHWRIQRLEARWFLDAYKARKDMNPIIYELGKIDFHIIQETQLQEVQEVSQWWTNTNLAEKLPFVRDRIVECYFWALGLFEPHEYGYQRKMAAIIITFVTIIDDVYDVYGTLDELQLFTDAIRKWDVESISTLPYYMQVCYLAVFTYASELAYDILKDQGFNSISYLQRSWLSLVEGFFQEAKWYYAGYTPTLAEYLENAKVSISSPTIISQVYFTLPNSTERTVVENVFGYHNILYLSGMILRLADDLGTTQFELKRGDVQKAIQCYMNDNNATEEEGTEHVKYLLREAWQEMNSAMADPDCPLSEDLVFAAANLGRASQFIYLDGDGHGVQHSEIHNQMGGLIFEPYV |
| TcTPS2.1 | MASLQVEEETRRSGNYQASIWDNDFIQSFNTNKYRDEKHLNRKEELIAQVKVLLNTKMEAVKQLELIDDLRNLGLTYYFQDEFKKILTCIYNDHKCFKNERVGDLYFTSLGFRLLRLHGFDVSEDVFSFFKNEDGSDFKASLGENTKDVLQLYEASFLVRVGEVTLEQARVFSTKILEKKVDEGINDEKLLAWIQHSLALPLHWRIQRLEARWFLDAYAARKDMNPLIFELGKIDFHIIQETQLEEVQEVSRWWTNSNLAEKLPFVRDRIVECYFWALGLFEPHEYGYQRKMAAIIITFVTIIDDVYDVYGTLDELQLFTDAIRKWDFESISTLPYYMQVCYLALYTYASELAYDILKDQGFNSISYLQRSWLSLVEGFFQEAKWYYAGYTPTLAEYLENAKVSISSPTIISQVYFTLPNSTERTVVENVYGYYNILYLSGMILRLADDLGTTQFELKRGDVQKAIQCYMKDNNATEKEGQEHVKYLLLEAWKEMNTAMADPDCPLSEDLVDAAANLGRASQFIYLEGDGHGVQHSEIHNQMGGLIFEPYV |
| TcTPS2.2 | MASLQVEEETRRSGNYQASIWDNAFIQSFNTNKYRDEKHLNRKEELIAQVKVLLNTKMEAVKQLELIDDLRNLGLTYYFQDEFKKILTCIYNDHKCFKNEQVGDLYFTSLGFRLLRLHGFDVSEEVFSFFKNEDGSDFKASLGENTKDVLQLYEASFLVRVGEVTLEQARVFSTKILEKKVDEGINDEKLLAWIQHSLALPLHWRIQRLEARWFLDAYAARKDMNPLIFELGKIDFHIIQETQLEEVQEVSRWWTNSNLAEKLPFVRDRIVECYFWALGLFEPHEYGYQRKMAAIIITFVTIIDDVYDVYGTLDELQLFTDAIRKWDFESISTLPYYMQVCYLALYTYASELAYDILKDQGFNSISYLQRSWLSLVEGFFQEAKWYYAGYTPTLAEYLENAKVSISSPTIISQVYFTLPNSTERTVVENVYGYHNILYLSGMILRLADDLGTTQFELKRGDVQKAIQCYMKDNNATEKEGQEHVKYLLLEAWKEMNTAMADPDCPLSEDLVDAAANLGRASQFIYLEGDGHGVQHSEIHNQMGGLIFEPYV |
| OvTPS2.1 | MATLSMQVSILSKEVKNVNNIGMRASKPMVARRVSTTRLRPICSASLQVEEETRRSGNYQASIWNNDYVQSFNTNQYKDEKHLKKKEELIAQVKILLNTKMEVVKQLELIEDLRNLGLTYYFQDEVKKILTSIYNDRKCFKNEQVGDLYFTSLGFRLLRLHGFDVSEEVFNFFKTENGSDFKASLGENIKDVLQLYEASFLIREGEVILEQARVFSTKHLEKKVDEGINDEKLLAWIRHSLALPLHWRIQRLEARWFLDAYKARKDMVPLIFELGKIDFHIIQETQLEELQEVSKWWTNSNLAEKLPFVRDRIVECYFWALGLFEPHEYGYQRKMAAIIITFVTIIDDVYDVYGTLDELQLFTDAIRKWDFQSISTLPYYMQVCYLALYTYASELAYDILKDQGFNSIAYLQRSWLSLVEGFFQEAKWYYAGYTPTLAEYLENAKVSISPPTIISQVYFTLPNSTERTVVENVFGYHNILYLSGMILRLADDLGTTQFELKRGDVQKAIQCYMKDNNATEKEGAEHVKYLLREAWKEMNTAMADPECPLSEDLVDAAANLGRASQFIYLEGDGHGVQHSEIHNQMGGLIFEPYV |
| OvTPS2.2 | MATLSMQVSILSKEVKNVNNIGMRASKPMVARRVSTTRLRPICSASLQVEEETRRSGNYQASIWNNDYVQSFNTNQYKDEKHLKKKEELIAQVKILLNTKMEAVKQLELIEDLRNLGLTYYFQDEVKKILTSIYNDHKCFKNEQVGDLYFTSLGFRLLRLHGFDVSEEVFDFFKNEDGSDFKASLGENIKDVLQLYEASFLIREGEVILEQARVFSTKHLEKKVDEGINDEKLLAWIRHSLALPLHWRIQRLEARWFLDAYRARKDMIPLIFELGKIDFHIIQETQLEELQEVSKWWTNSNLAEKLPFVRDRIVECYFWALGLFEPHEYGYQRKMAAIIITFVTIIDDVYDVYGTLDELQLFTDAIRKWDFQSISTLPYYMQVCYLALYTYASELAYDILKDQGFNSIAYLQRSWLSLVEGFFQEAKWYYAGYTPTLAEYLENAKVSISSPTIISQVYFTLPNSTERTVVENVFGYHNILYLSGMILRLADDLGTTQFELKRGDVQKAIQCYMKDNNATEKEGAEHVKYLLREAWKEMNTAMADPECPLSEDLVDAAANLGRASQFIYLEGDGHGVQHSEIHNQMGGLIFEPYV |
| PaTPS-Lon | MAQISKCSSLSAELNESSIISHHHGNLWDDDFIQSLKSSNGAPQYHERAAKLVEEIKNLVVSEMKDCNDDLIRRLQMVDIFECLGIDRHFQHEIQVALDYVYRYWNQLEGIGIGSRDSLIKDFNATALGFRALRLHRYNVSSDVLENFKNENGQFFCSSTVEEKEVRCMLTLFRASEISFPGEKVMDEAKAFTTEYLTKVLTGVDVTDVNQSLLREVKYALEFPWHCSLPRWEARSFIEICGQNDSWLKSIMNKRVLELAKLDFNILQWAHHRELQLLSSWWSQSDIAQQNFYRKRHVEFYLWVVIGTFEPEFSTCRITFAKISTLMTILDDLYDTHGTLEQLKIFTEGVKRWDLSLVDRLPDYIKITFEFFLNTSNELIAEVAKTQERDMSAYIRKTWERYLEAYLQEAEWIAARHVPTFDEYMKNGISSSGMCILNLYSLLLMGQLLPDDVLEQIHSPSKIHELVELTARLVDDSKDFETKKVGGELASGIECYVKDNPECTLEDASNHLNGLLDLTVKELNWEFVRHDSVALCFKKFAFNVARGLRLIYKYRDGFDVSNQEMKTHIFKILIDPLT |

**Table S8 Primers sequences**

| **Name** | **Sequence** |
| --- | --- |
| pMAL-TPS1-F | GAGGGAAGGATTTCAGAATTCATGTATAGCATCAGCAGTAACGTG |
| pMAL-TPS1-R | CAAGCTTGCCTGCAGGTCGACTCAGATGTATGGCTCCAACATGAG |
| pMAL-TPS2-F | GAGGGAAGGATTTCAGAATTCATGTGTAGCATGAGAATACATGT |
| pMAL-TPS2-R | CAAGCTTGCCTGCAGGTCGACTTAATCTCCGATTTCTGAGGTA |
| pMAL-TPS3-F | GAGGGAAGGATTTCAGAATTCATGTGTAGCATGAGAATACATGT |
| pMAL-TPS3-R | CAAGCTTGCCTGCAGGTCGACTCATGCATATGGCTCGAACAGCAGAC |
| pMAL-TPS4-F | GAGGGAAGGATTTCAGAATTCATGCGTAGCGTTTTATTGCAAGTG |
| pMAL-TPS4-R | CAAGCTTGCCTGCAGGTCGACTTAGGCATAGCGGTCGAACAGCAAG |
| pMAL-TPS5-F | GAGGGAAGGATTTCAGAATTCATGTCTAGCATTATACTAATGCATAC |
| pMAL-TPS5-R | CAAGCTTGCCTGCAGGTCGACCTAGACAATAGGGTTGAATATCA |
| pHER-McCYP71D179-F | TATATTAAACGTCTCTAAAAATGTACTCAATGGAGCTCAATACTCTC |
| pHER-McCYP71D179-R | AATTTAATGAAACCAGAGCGTTAAGGATGAGTGGGAGTGTAGATG |
| pHER-McSDR2-F | TATATTAAACGTCTCTAAAAATGTCAAAACCAAGGTTGGAG |
| pHER-McSDR2-R | AATTTAATGAAACCAGAGCGTCAAAACGAACTGGAAGAGTAAC |
| pTRV2-McPDS-EcoRI-F | GTGAGTAAGGTTACCGAATTCGACAGTGAGCAAATCTGTGC |
| pTRV2-McPDS-BamHI-R | GTGAGCTCGGTACCGGATCCGATCTGGTTTGGTGTCGTGG |
| pTRV2-McTPS1-EcoRI-F | GTGAGTAAGGTTACCGAATTCTATAGCATCAGCAGTAACGTGAA |
| pTRV2-McTPS1-BamHI-R | GTGAGCTCGGTACCGGATCCCAGCATCAGCTTCACTTGCTCA |
| pTRV2-McCYP71D179-EcoRI-F | GTGAGTAAGGTTACCGAATTCGCTGTCGGCTCTGTCGGCGCAG |
| pTRV2-McCYP71D179-BamHI-R | GTGAGCTCGGTACCGGATCCCTCTCCTAGGCGTCCTCTTCTCA |
| pTRV2-McSDR2-EcoRI-F | GTGAGTAAGGTTACCGAATTCCATCCGACGCAAGAAACAGC |
| pTRV2-McSDR2-BamHI-R | GTGAGCTCGGTACCGGATCCGGTCCATCATCTGCACCGCCAG |
| pB42AD-EcoRI-McMIXTA-F | GCCTCTCCCGAATTCATGGGCCGGTCTCCTTGTTGC |
| pB42AD-XhoI-McMIXTA-R | CCAAAGCTTCTCGAGAAATATTGGTGAATCAGAGGGCGAAG |
| pB42AD-XhoI-McWIN1-R | CCAAAGCTTCTCGAGATTCCTGTTTAGAAGCTCCTCGATC |
| proMcTPS1-MBS1-E/X-F | aattcGGGCGAAGGATTTGTGTTGGTGGTTGGTGGTTGGTGCACTCAAGAAATTAATc |
| proMcTPS1-MBS1-E/X-R | tcgagATTAATTTCTTGAGTGCACCAACCACCAACCACCAACACAAATCCTTCGCCCg |
| proMcTPS1-MBS2-E/X-F | aattcAAAATTAATAAAAGCAATAACGGTAACGGTAACGGTACACTATGTGGGATATc |
| proMcTPS1-MBS2-E/X-R | tcgagATATCCCACATAGTGTACCGTTACCGTTACCGTTATTGCTTTTATTAATTTTg |
| proMcCYP71D179-MBS1-E/X-F | aattcTATTTAAAAAAAATCACCTACCCACCTACCCACCTACCCTACTTACAAAACAc |
| proMcCYP71D179-MBS1-E/X-R | tcgagTGTTTTGTAAGTAGGGTAGGTGGGTAGGTGGGTAGGTGATTTTTTTTAAATAg |
| proMcCYP71D179-MBS2-E/X-F | aattcAATGCCTAAGAAACTACCTACTACCTACTACCTACTTCTAAATATTGTCGc |
| proMcCYP71D179-MBS2-E/X-R | tcgagCGACAATATTTAGAAGTAGGTAGTAGGTAGTAGGTAGTTTCTTAGGCATTg |
| proMcCYP71D179-HD-E/X-F | aattcGCAAATTGTAATCGTACATTTATACATTTATACATTTATTTATCAAAATAAAc |
| proMcCYP71D179-HD-E/X-R | tcgagTTTATTTTGATAAATAAATGTATAAATGTATAAATGTACGATTACAATTTGCg |
| proMcCYP71D179-GCC-E/X-F | aattcATAGACCACAAATTCTCGGCGGCGGCGGCGGCGGCTGACTCAGAATTCAATAc |
| proMcCYP71D179-GCC-E/X-R | tcgagTATTGAATTCTGAGTCAGCCGCCGCCGCCGCCGCCGAGAATTTGTGGTCTATg |
